# Sitagliptin ameliorates radiation-induced oral mucositis by inhibiting oxidative damage and inflammatory response via TLR2/MyD88/NF-κB signaling pathway

**DOI:** 10.1186/s43556-026-00653-8

**Published:** 2026-11-09

**Authors:** Yongbiao Huang, Qingqing Yu, Yuanmei Yan, Duo Xu, Jingyao Tu, Yuting Huang, Rong Long, Meng Lyu, Bo Liu, Wan Qin, Xianglin Yuan

**Affiliations:** Department of Oncology, Tongji Hospital, Tongji Medical College, Huazhong University of Science and Technology, Wuhan, Hubei 430030 China

**Keywords:** Radiation-induced oral mucositis, Sitagliptin, Oxidative stress, Inflammatory response, TLR2/MyD88/NF-κB pathway

## Abstract

Radiotherapy for head and neck cancers is frequently complicated by radiation-induced oral mucositis (RIOM), which can compromise treatment tolerance and negatively affect patients’ quality of life. Current preventive and therapeutic options remain limited, highlighting the need for safer and more effective interventions. The development of RIOM involves a cascade of radiation-triggered oxidative stress, epithelial injury, and sustained inflammatory responses. Given the reported antioxidant and anti-inflammatory activities of sitagliptin, a widely used dipeptidyl peptidase − 4 inhibitor, we investigated its potential protective effects against RIOM. Here we show that oral sitagliptin markedly reduces mucosal ulceration and radiation-associated weight loss in mouse models of localized head-and-neck irradiation, while preserving mucosal thickness and basal epithelial stem cells and sustaining proliferative activity after irradiation. Sitagliptin limits reactive oxygen species accumulation and lipid peroxidation, restores glutathione levels and superoxide dismutase activity, and reduces DNA damage, apoptosis, inflammatory cytokine expression, and inflammatory-cell infiltration. Mechanistically, sitagliptin inhibits TLR2/MyD88/NF-κB signaling, whereas pharmacological activation of TLR2 partially reverses its protective effects in vitro, providing further evidence that this pathway contributes to sitagliptin-mediated protection. Sitagliptin is well tolerated in healthy mice, causes no detectable hypoglycemia or organ toxicity, and does not measurably impair radiotherapy-mediated tumor control or tumor radiosensitivity. Collectively, these results demonstrate that sitagliptin protects against RIOM through coordinated antioxidant, anti-apoptotic, and anti-inflammatory actions involving suppression of TLR2/MyD88/NF-κB signaling, while maintaining tumor radiosensitivity. Its established clinical use and the efficacy and tolerability observed in our preclinical models support further evaluation of sitagliptin for RIOM.

**Supplementary Information:**

The online version contains supplementary material available at https://doi.org/10.1186/s43556-026-00653-8.

## Introduction

Although radiotherapy is a key treatment for head and neck cancers, it can also damage surrounding healthy tissues, leading to treatment-related complications and limiting the dose that can be safely delivered [1]. Radiation-induced oral mucositis (RIOM) is the most prevalent form of radiotoxicity, occurring in nearly all patients undergoing treatment for head and neck malignancies [2]. Clinically, RIOM is characterized by intense pain, mucosal erythema, and ulcer formation, often resulting in dysphagia, nutritional deficiencies, and secondary infections. These complications frequently necessitate treatment interruptions or dose reductions [3, 4], thereby undermining tumor control and adversely affecting survival outcomes. Current management strategies for RIOM are largely limited to symptomatic relief [5, 6], highlighting the need to develop safe and effective preventive interventions.

The pathogenesis of RIOM is generally conceptualized as a multifactorial and dynamic process consisting of five phases: initiation, primary damage, signaling amplification, ulceration, and healing [7, 8]. During the initiation phase, ionizing radiation rapidly increases reactive oxygen species (ROS) generation and causes DNA double-strand breaks (DSBs). This initial oxidative damage triggers NF-κB signaling pathway activation and promotes the secretion of pro-inflammatory cytokines such as IL-6, IL-1β, and TNF-α. These cytokines further amplify inflammatory signaling through positive feedback mechanisms, intensifying NF-κB activity during the amplification phase. Sustained oxidative stress and inflammation ultimately result in epithelial breakdown and basement membrane disruption, culminating in ulcer formation [9, 10]. Consequently, early elimination of ROS and inhibition of NF-κB-driven inflammatory cascades are considered critical targets for RIOM prevention and treatment.

Sitagliptin is an orally administered dipeptidyl peptidase − 4 (DPP-4) inhibitor widely used for the treatment of type 2 diabetes mellitus [11–13]. Beyond glucose control, previous studies have shown that sitagliptin also possesses anti-inflammatory, antioxidant, and anti-apoptotic properties [14–17]. In the context of radioprotection, sitagliptin has shown efficacy in safeguarding the hematopoietic system and reducing radiation-induced injury in the intestine and kidneys [18–21]. These protective effects are largely attributed to its ability to regulate redox balance and suppress inflammatory signaling pathways. Evidence from earlier studies suggests that sitagliptin can scavenge free radicals, reduce oxidative byproducts, and suppress pro-inflammatory cytokine expression [16, 22]. Such pleiotropic properties provide a solid theoretical basis for using sitagliptin to intervene in the early oxidative stress and signaling amplification phases of RIOM.

Despite these promising observations, the effects of sitagliptin on RIOM and the associated molecular mechanisms remain incompletely understood. In this study, we investigated the protective effects of sitagliptin in mouse models of RIOM induced by single-dose irradiation and fractionated irradiation, and explored the underlying mechanisms. Additionally, we assessed the systemic safety of sitagliptin and whether it affects the antitumor efficacy of radiotherapy. The overall goal of this study was to provide preclinical evidence supporting the potential clinical use of sitagliptin in RIOM management.

## Results

### Sitagliptin markedly reduces radiation-induced oral mucosal injury in mice

**Fig. 1:**
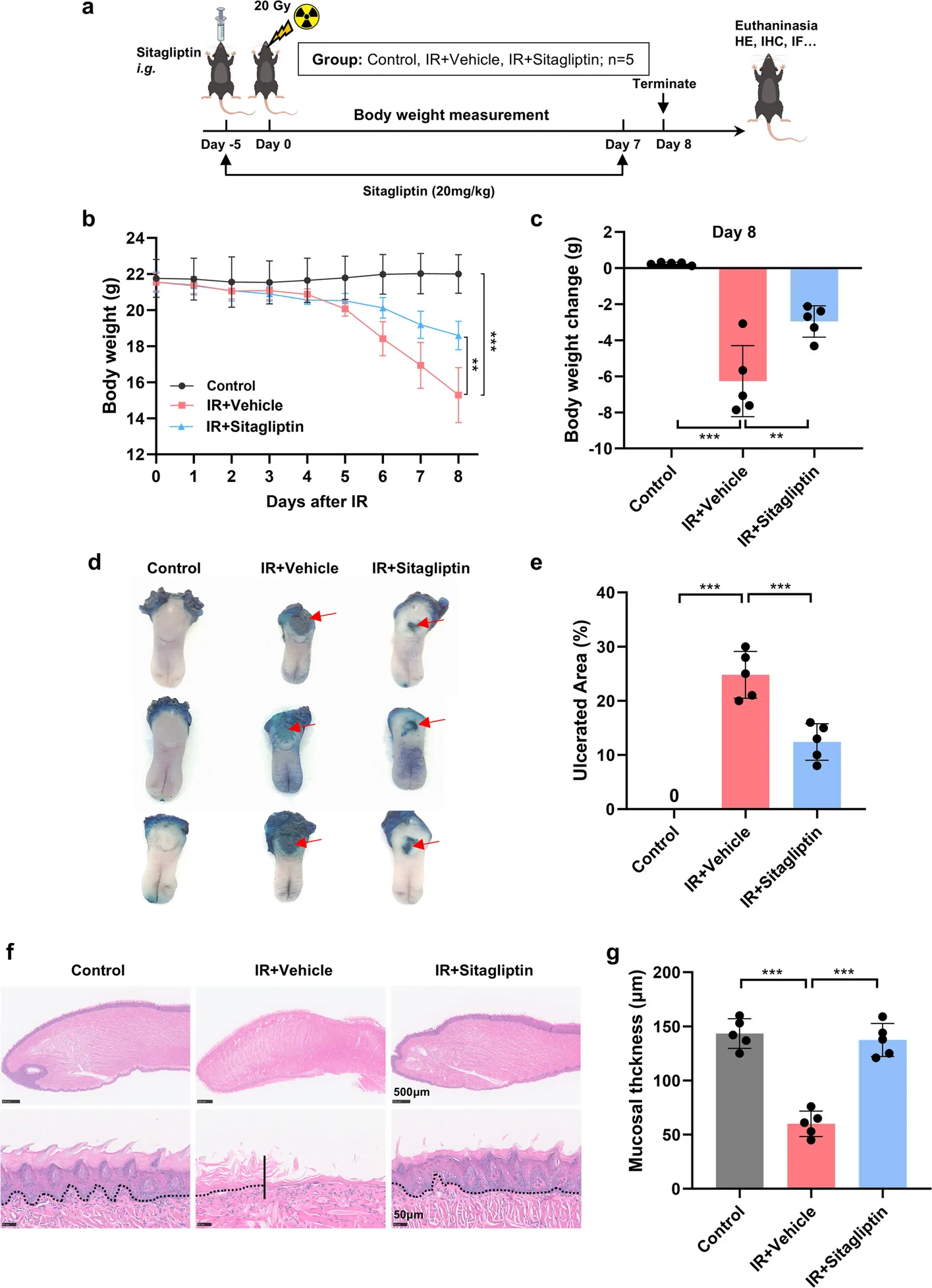
Sitagliptin mitigates RIOM in mice following single-dose irradiation. **a** Experimental schematic of single-dose 20 Gy irradiation. **b** Body weight changes following irradiation. **c** Body weight change on day 8 post-irradiation. **d** Representative images of toluidine blue-stained tongues at day 8; red arrows denote ulcerative lesions. **e** Quantification of ulcerated surface area. **f** H&E staining of mouse tongue mucosa on day 8 following irradiation. The vertical lines in images from the IR + Vehicle group indicate ulcer boundaries, whereas dotted lines indicate the epithelial–stromal boundary. **g** Quantification of mucosal thickness. Data are presented as mean ± SD (*n* = 5). *P < 0.05, **P < 0.01, ***P < 0.001

As illustrated in Fig. 1a, a murine RIOM model was established using single-dose 20 Gy irradiation, and body weight was monitored throughout the experimental period. Mice in the IR + Vehicle group began to exhibit significant weight loss by day 5, progressing to pronounced overall weight reduction by day 8. In contrast, treatment with sitagliptin significantly attenuated this radiation-associated weight loss (Fig. 1b, c). Gross examination of the tongue revealed that, while the Control group maintained intact mucosa, mice subjected to irradiation developed extensive ulcerative lesions on the mucosal surface. Quantitative assessment demonstrated that sitagliptin treatment significantly decreased the proportion of ulcerated area compared with the IR + Vehicle group (Fig. 1d, e). Histopathological evaluation using H&E staining showed that irradiation caused severe epithelial disruption and mucosal atrophy, leading to a marked reduction in mucosal thickness. In contrast, sitagliptin preserved mucosal architecture and significantly increased mucosal thickness relative to irradiated controls (Fig. 1f, g).

To further evaluate the protective effect of sitagliptin under fractionated irradiation, we established a fractionated RIOM model in which mice received 8 Gy once daily for three consecutive days (8 Gy × 3; total dose, 24 Gy). Fractionated irradiation caused marked oral mucosal injury, as evidenced by body weight loss, increased ulcerated area, and reduced mucosal thickness. Compared with the IR + Vehicle group, sitagliptin treatment significantly attenuated radiation-associated body weight loss, reduced mucosal ulceration, and preserved mucosal thickness (Fig. S1). Collectively, these findings indicate that sitagliptin provides substantial protection against radiation-induced oral mucosal damage under both single-dose and fractionated irradiation conditions.

### Sitagliptin preserves basal epithelial stem/progenitor cells and sustains proliferative activity after irradiation

**Fig. 2:**
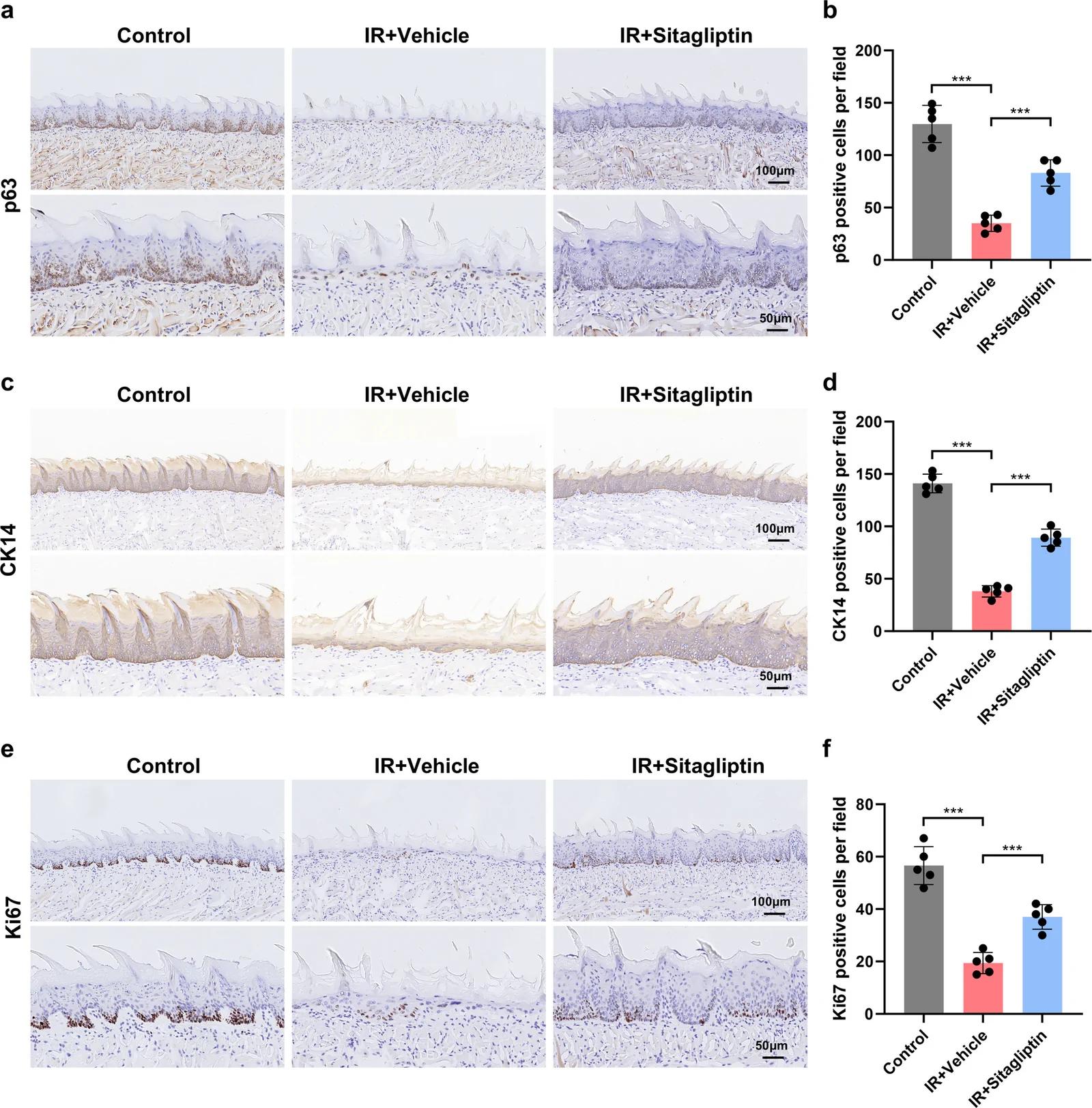
Sitagliptin preserves basal epithelial stem/progenitor cells and maintains the proliferative capacity of the irradiated oral mucosa. **a**, **b** Representative IHC staining and quantification of p63-positive cells. **c**, **d** IHC staining and quantification of CK14-positive cells. **e**, **f** IHC staining and quantification of Ki67-positive cells in tongue mucosal tissues. Data are presented as mean ± SD (*n* = 5). ***P < 0.001

Maintenance of oral mucosal integrity depends on the continuous renewal of basal epithelial stem/progenitor cells. To evaluate this regenerative compartment, the expression of p63 and cytokeratin 14 (CK14), two markers associated with basal epithelial stem/progenitor cells, together with the proliferation marker Ki67, was assessed. As displayed in Fig. 2a and b, irradiation led to a significant reduction in the number of p63-positive basal epithelial cells compared with the Control group. This loss was markedly attenuated by sitagliptin treatment. Consistently, CK14-positive basal epithelial cells were also markedly reduced following irradiation and were significantly preserved by sitagliptin treatment (Fig. 2c, d). Similarly, the number of Ki67-positive proliferating cells in the basal layer was substantially decreased following irradiation, indicating impaired proliferative capacity. Sitagliptin administration effectively restored the population of proliferating cells (Fig. 2e, f).

Together, the concordant changes in p63 and CK14, along with the preservation of Ki67-positive cells, indicate that sitagliptin helps maintain the basal epithelial stem/progenitor compartment and regenerative capacity after radiation exposure.

### Sitagliptin reduces radiation-induced oxidative stress, DNA damage, and apoptosis

**Fig. 3:**
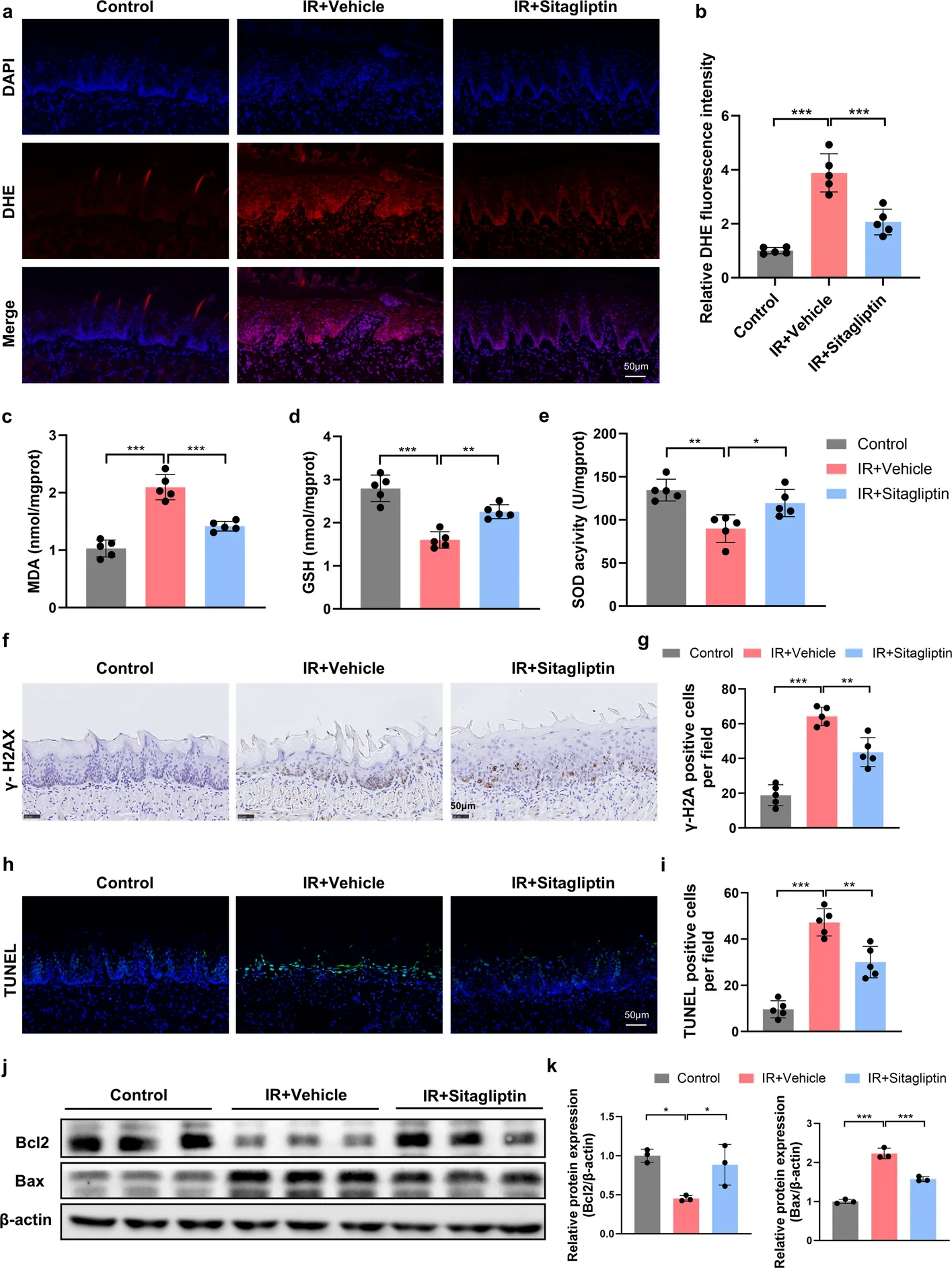
Sitagliptin attenuates oxidative stress, DNA damage, and apoptosis in the irradiated oral mucosa. **a**, **b** DHE staining and quantification of relative fluorescence intensity (*n* = 5). **c**–**e** Measurements of MDA, GSH levels and SOD activity in tongue tissues (*n* = 5). **f**, **g** IHC staining and quantification of γ-H2AX-positive cells (*n* = 5). **h**, **i** TUNEL staining and quantification of TUNEL-positive cells (*n* = 5). **j**, **k** Western blot analysis and quantification of Bcl-2 and Bax protein expression (*n* = 3). Data are presented as mean ± SD. *P < 0.05, **P < 0.01, ***P < 0.001

Excessive oxidative stress is a key initiating factor in radiation-induced mucosal injury. DHE fluorescence staining revealed a marked increase in ROS levels in irradiated tissues, whereas sitagliptin treatment significantly reduced ROS accumulation (Fig. 3a, b). Biochemical analyses further demonstrated that sitagliptin reversed the radiation-induced elevation of the lipid peroxidation marker MDA, while restoring GSH levels and SOD activity (Fig. 3c–e). The overproduction of ROS contributes to DNA DSBs. Immunohistochemical analysis showed a substantial increase in γ-H2AX-positive cells following irradiation, indicative of DNA damage, whereas this effect was significantly mitigated by sitagliptin (Fig. 3f, g). Persistent DNA damage can activate apoptotic pathways. TUNEL staining demonstrated a pronounced accumulation of apoptotic cells in the irradiated mucosa, which was significantly reduced with sitagliptin treatment (Fig. 3h, i). Consistent with these findings, Western blotting further showed that sitagliptin restored Bcl-2 expression reduced by irradiation and suppressed the radiation-associated elevation of Bax (Fig. 3j, k). Collectively, these findings show that sitagliptin attenuates radiation-associated oxidative stress, DNA damage, and apoptosis in oral mucosal tissues.

### Sitagliptin attenuates radiation-induced inflammatory responses in the oral mucosa

**Fig. 4:**
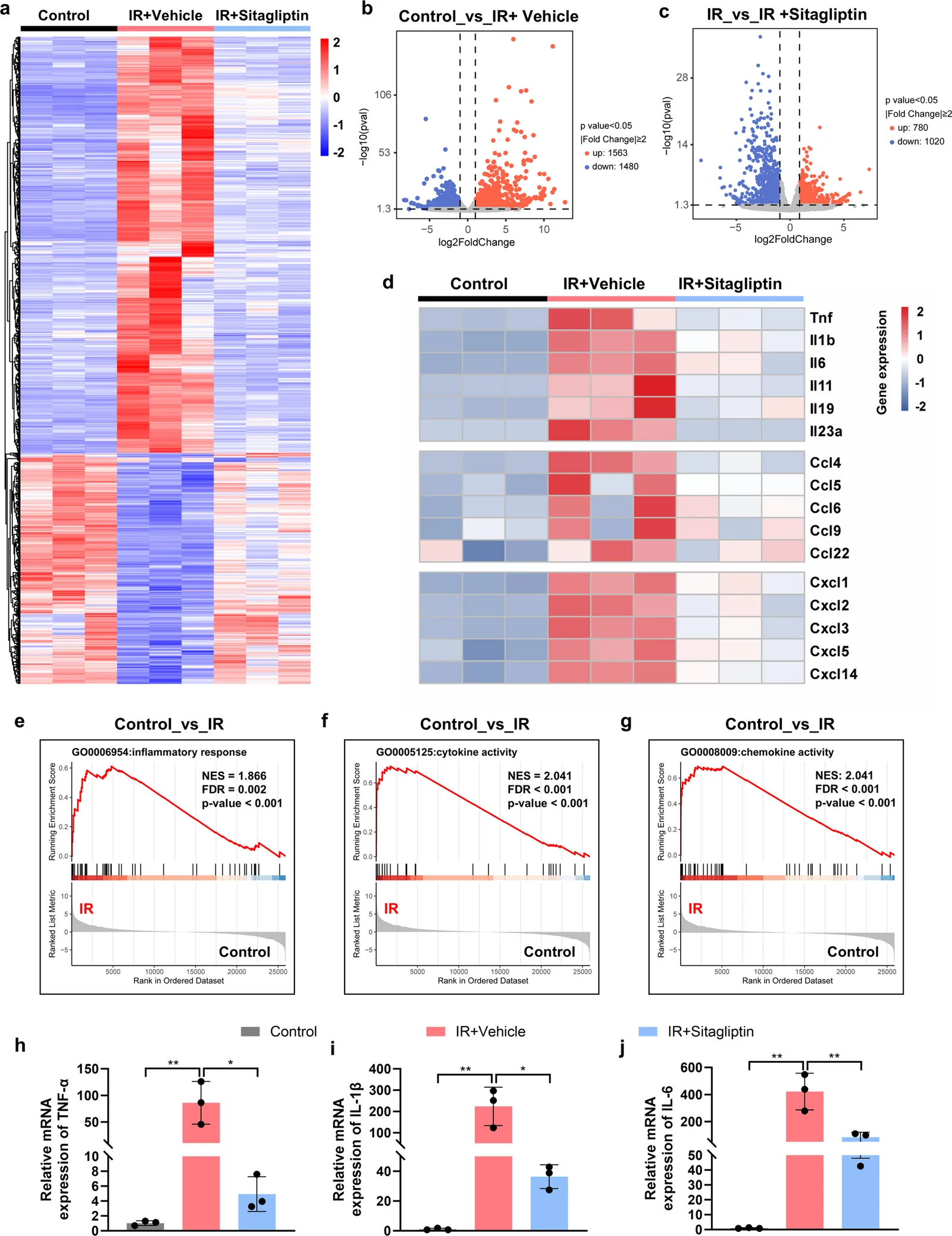
Sitagliptin reverses radiation-induced transcriptomic alterations and suppresses mucosal inflammatory responses. **a** Heatmap showing hierarchical clustering of DEGs. **b**, **c** Volcano plots showing DEGs between Control and IR + Vehicle groups (1563 upregulated and 1480 downregulated genes) and between IR + Vehicle and IR + Sitagliptin groups (780 upregulated and 1020 downregulated genes), respectively. **d** Heatmap of pro-inflammatory cytokine and chemokine expression profiles, including Tnf, Il1b, Il6, Il11, Il9, Il23a, Ccl4, Ccl5, Ccl6, Ccl9, Ccl22, Cxcl1, Cxcl2, Cxcl3, Cxcl5, Cxcl14. **e**–**g** GSEA plots showing enrichment of inflammatory response (NES = 1.866, FDR = 0.002, *p*-value < 0.001), cytokine activity (NES = 2.041, FDR < 0.001, *p*-value < 0.001), and chemokine activity (NES = 2.041, FDR < 0.001, *p*-value < 0.001) in irradiated tissues. **h**–**j** qPCR validation of TNF-α, IL-1β, and IL-6 expression levels (*n* = 3), data are presented as mean ± SD. *P < 0.05, **P < 0.01

To explore the molecular mechanisms of sitagliptin-mediated mucosal protection, transcriptomic profiling was performed on mouse tongue tissues. Principal component analysis (PCA) demonstrated that samples from the sitagliptin-treated group shifted toward the clustering pattern of the control group, indicating partial restoration of the radiation-altered transcriptome (Fig. S2a). Differential expression analysis further revealed that irradiation markedly disrupted gene expression patterns in mucosal tissues (Fig. 4a–c and Fig. S2b). Venn diagram comparisons showed that sitagliptin treatment reversed a subset of these radiation-induced transcriptional alterations (Fig. S2c–e).

Heatmap visualization of inflammation-associated genes indicated that irradiation significantly elevated the expression of multiple pro-inflammatory cytokines and chemokines, whereas sitagliptin markedly reduced their transcriptional levels (Fig. 4d). To further investigate the biological processes involved, gene set enrichment analysis (GSEA) was conducted. The results showed that pathways related to inflammation, including inflammatory response, cytokine activity, and chemokine activity, were significantly enriched following irradiation (Fig. 4e–g). In contrast, these pathways were notably suppressed in the sitagliptin-treated group (Fig. S2f–h). Consistent with the sequencing data, qPCR validation confirmed that sitagliptin attenuated the irradiation-associated increases in TNF-α, IL-1β, and IL-6 mRNA levels (Fig. 4h–j). To further characterize inflammatory-cell infiltration in the irradiated oral mucosa, immunofluorescence staining was performed for F4/80, MPO, and CD3. Irradiation markedly increased the infiltration of F4/80-positive macrophages, MPO-positive neutrophils, and CD3-positive T cells, whereas these increases were significantly reduced by sitagliptin treatment (Fig. S3). Together, these findings demonstrate that sitagliptin attenuates radiation-induced inflammatory responses in the oral mucosa.

### Sitagliptin attenuates radiation-induced inflammation and suppresses TLR2/MyD88/NF-κB signaling

**Fig. 5:**
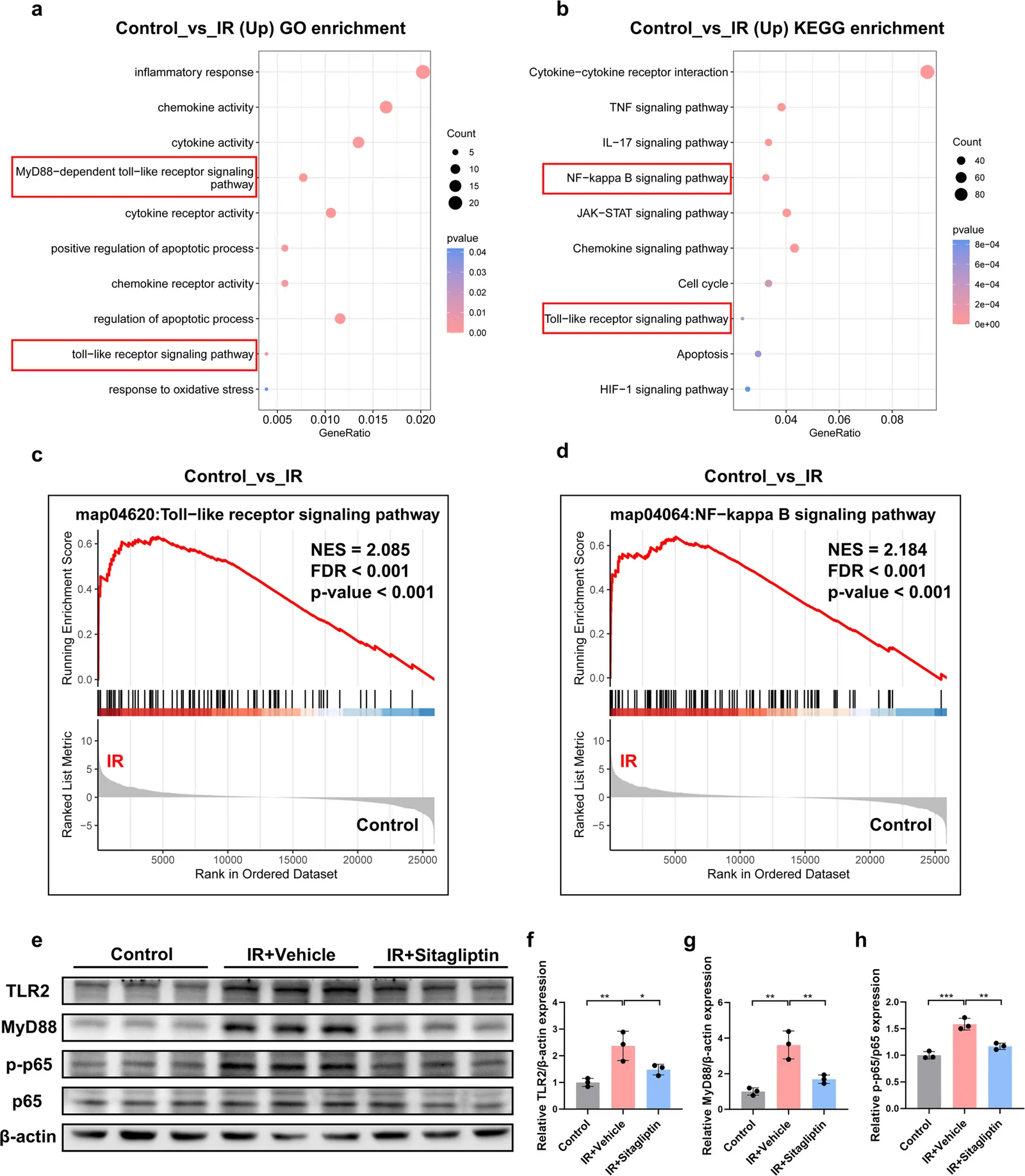
Sitagliptin suppresses activation of the TLR2/MyD88/NF-κB signaling pathway induced by radiation. **a** GO enrichment analysis of upregulated DEGs in irradiated tissues. Red boxes highlight the MyD88-dependent Toll-like receptor signaling pathway (GeneRatio = 0.008, Count = 8, *p*-value = 2.62E − 08) and Toll-like receptor signaling pathway (GeneRatio = 0.004, Count = 4, *p*-value = 0.003). **b** KEGG enrichment analysis of upregulated DEGs in irradiated tissues. Red boxes highlight the NF-κB signaling pathway (GeneRatio = 0.052, Count = 33, *p*-value = 4.42E − 13) and Toll-like receptor signaling pathway (GeneRatio = 0.034, Count = 22, *p*-value = 3.07E − 06). **c**, **d** GSEA showing significant enrichment of the Toll-like receptor signaling pathway (NES = 2.085, FDR < 0.001, *p*-value < 0.001) and NF-κB signaling pathway (NES = 2.184, FDR < 0.001, *p*-value < 0.001) in irradiated tissues. **e**–**h** Western blotting and quantitative analysis of TLR2, MyD88, p-p65, and p65 protein expression (*n* = 3). Data are presented as mean ± SD. *P < 0.05, **P < 0.01, ***P < 0.001

To identify upstream regulatory mechanisms driving the observed inflammatory changes, further bioinformatics analyses were conducted. GO and KEGG enrichment analyses indicated that irradiation induced Toll-like receptor (TLR) signaling and NF-κB pathway upregulation (Fig. 5a, b). These pathways are firmly established as central mediators of inflammation, detecting tissue damage and promoting the release of pro-inflammatory cytokines [23, 24]. In contrast, sitagliptin treatment significantly suppressed the activation of these signaling cascades, as evidenced by their downregulation (Fig. S4a, b). GSEA provided additional support for these observations, showing pronounced activation of the TLR and NF-κB pathways in irradiated tissues (Fig. 5c, d), while they were markedly suppressed following sitagliptin administration (Fig. S4c, d).

To validate these transcriptomic findings at the protein level, key components of the pathway were examined. Western blotting revealed that irradiation significantly increased the expression of TLR2 and the adaptor protein MyD88, along with enhanced phosphorylation of NF-κB p65 (p-p65) in mucosal tissues (Fig. 5e). Quantitative analysis demonstrated that sitagliptin significantly reduced the expression of TLR2 and MyD88 and decreased the p-p65/p65 ratio (Fig. 5f–h). Together, these findings demonstrate that the anti-inflammatory effects of sitagliptin are associated with suppression of TLR2/MyD88/NF-κB signaling, providing the basis for further investigation of the functional involvement of this pathway in its protective effects.

### Pharmacological activation of TLR2 signaling attenuates the protective effects of sitagliptin against radiation-induced epithelial cell injury

**Fig. 6:**
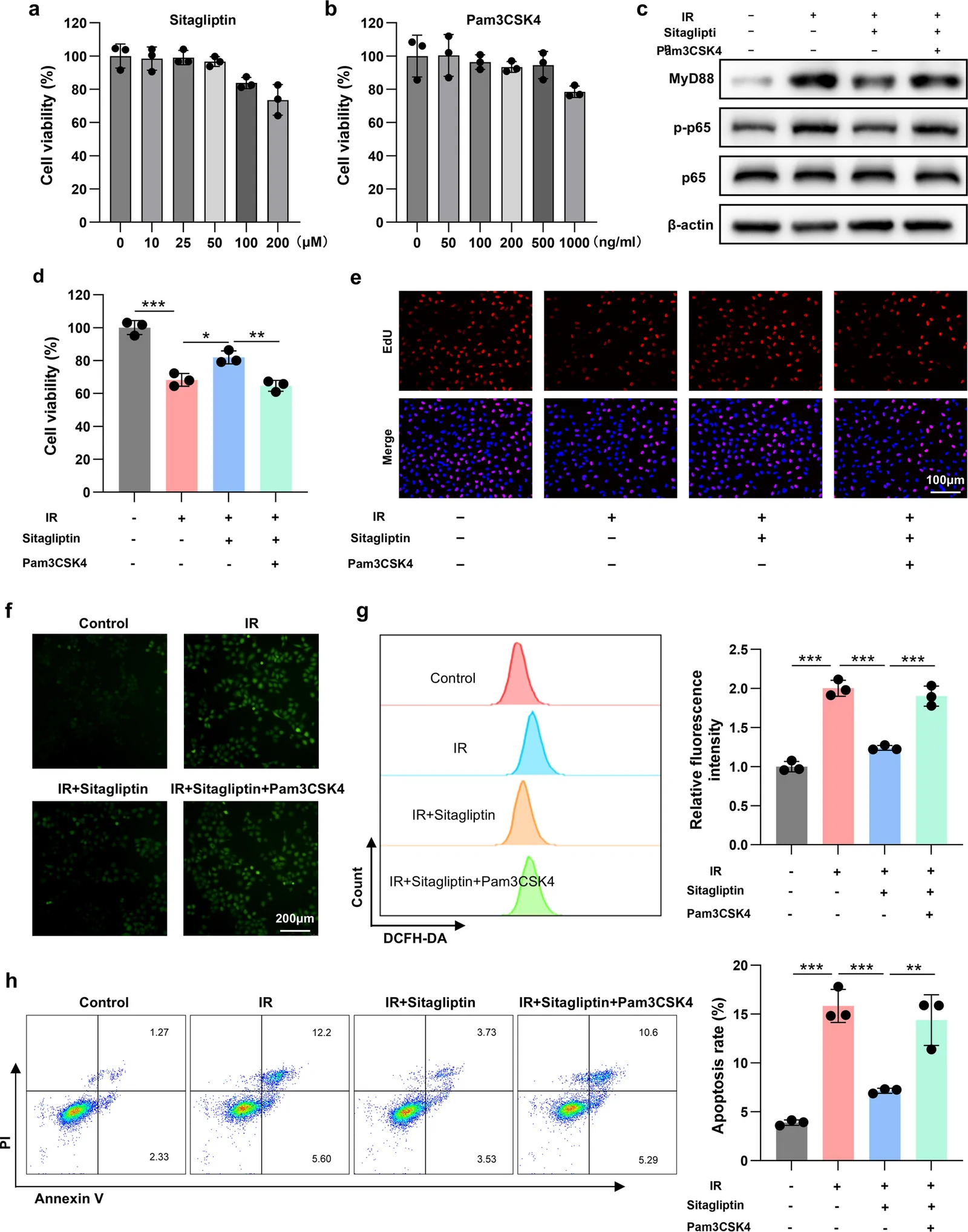
Pharmacological activation of TLR2 signaling attenuates the protective effects of sitagliptin against radiation-induced epithelial cell injury. **a**, **b** CCK-8 assays assessing the effects of different concentrations of sitagliptin and Pam3CSK4 on HOK cell viability after 24 h. **c** Western blot analysis of MyD88, p-p65, and p65. **d** CCK-8 assay of cell viability. **e** EdU assay. **f**, **g** Intracellular ROS detection by fluorescence microscopy and flow cytometry. **h** Cell apoptosis assessed by Annexin V-FITC/PI flow cytometry. Data are presented as mean ± SD from three independent experiments. *P < 0.05, **P < 0.01, ***P < 0.001

To further investigate the involvement of TLR2/MyD88/NF-κB signaling in the protective effects of sitagliptin, rescue experiments were performed in irradiated human oral keratinocytes (HOK) using Pam3CSK4, a TLR1/2 agonist, to pharmacologically activate TLR2 signaling. Preliminary CCK-8 assays showed that 24 h treatment with sitagliptin (up to 50 μM) or Pam3CSK4 (up to 500 ng/mL) did not significantly affect HOK cell viability under non-irradiated conditions (Fig. 6a, b). Based on these results and preliminary evaluation of their protective or pathway-activating effects, 50 μM sitagliptin and 500 ng/mL Pam3CSK4 were selected for subsequent experiments.

Western blotting showed that irradiation enhanced MyD88 expression and NF-κB p65 phosphorylation, whereas sitagliptin attenuated these changes. Notably, co-treatment with Pam3CSK4 reactivated downstream MyD88/NF-κB signaling in sitagliptin-treated irradiated HOK cells (Fig. 6c). Irradiation markedly reduced cell viability and EdU incorporation while increasing intracellular ROS accumulation and apoptosis, all of which were significantly alleviated by sitagliptin. However, Pam3CSK4 significantly attenuated these protective effects, as evidenced by reduced cell viability and EdU incorporation, together with increased ROS accumulation and apoptosis, compared with the IR + Sitagliptin group (Fig. 6d–h). Furthermore, sitagliptin significantly attenuated the irradiation-associated increases in TNF-α, IL-1β, and IL-6 mRNA levels, whereas co-treatment with Pam3CSK4 significantly reversed these inhibitory effects (Fig. S5).

Together, these rescue experiments show that reactivation of TLR2/MyD88/NF-κB signaling attenuates the protective effects of sitagliptin, supporting the involvement of this pathway in sitagliptin-mediated protection against radiation-induced epithelial cell injury.

### Sitagliptin demonstrates favorable in vivo safety

To assess the systemic safety profile of sitagliptin, a comprehensive in vivo toxicity evaluation was performed. During the 8-day observation period, mice receiving sitagliptin exhibited body weight trends comparable to those of control animals, with no statistically significant differences observed (Fig. S6a). Given the glucose-lowering action of sitagliptin, fasting blood glucose levels were monitored in healthy mice, and no hypoglycemia or marked glycemic fluctuations were observed (Fig. S6b). Serum biochemical analyses showed comparable levels of alanine aminotransferase (ALT), aspartate aminotransferase (AST), albumin (ALB), blood urea nitrogen (BUN), and creatinine (CREA) between the sitagliptin-treated and control groups, indicating no detectable impairment of hepatic or renal function (Fig. S6c–g). Histopathological examination further revealed no apparent treatment-related abnormalities in the heart, liver, spleen, lungs, or kidneys (Fig. S6h).

To further assess the safety of prolonged sitagliptin administration and potential delayed toxicity, an additional 30-day safety evaluation was performed. No significant differences were observed between the sitagliptin-treated and control groups in body weight, fasting blood glucose, or serum biochemical parameters, and no apparent histopathological abnormalities were detected in the major organs examined (Fig. S7). Collectively, these findings indicate that sitagliptin was well tolerated, with no detectable systemic toxicity under the short-term and prolonged treatment conditions evaluated in this study.

### Sitagliptin does not impair the antitumor effects of radiotherapy

**Fig. 7:**
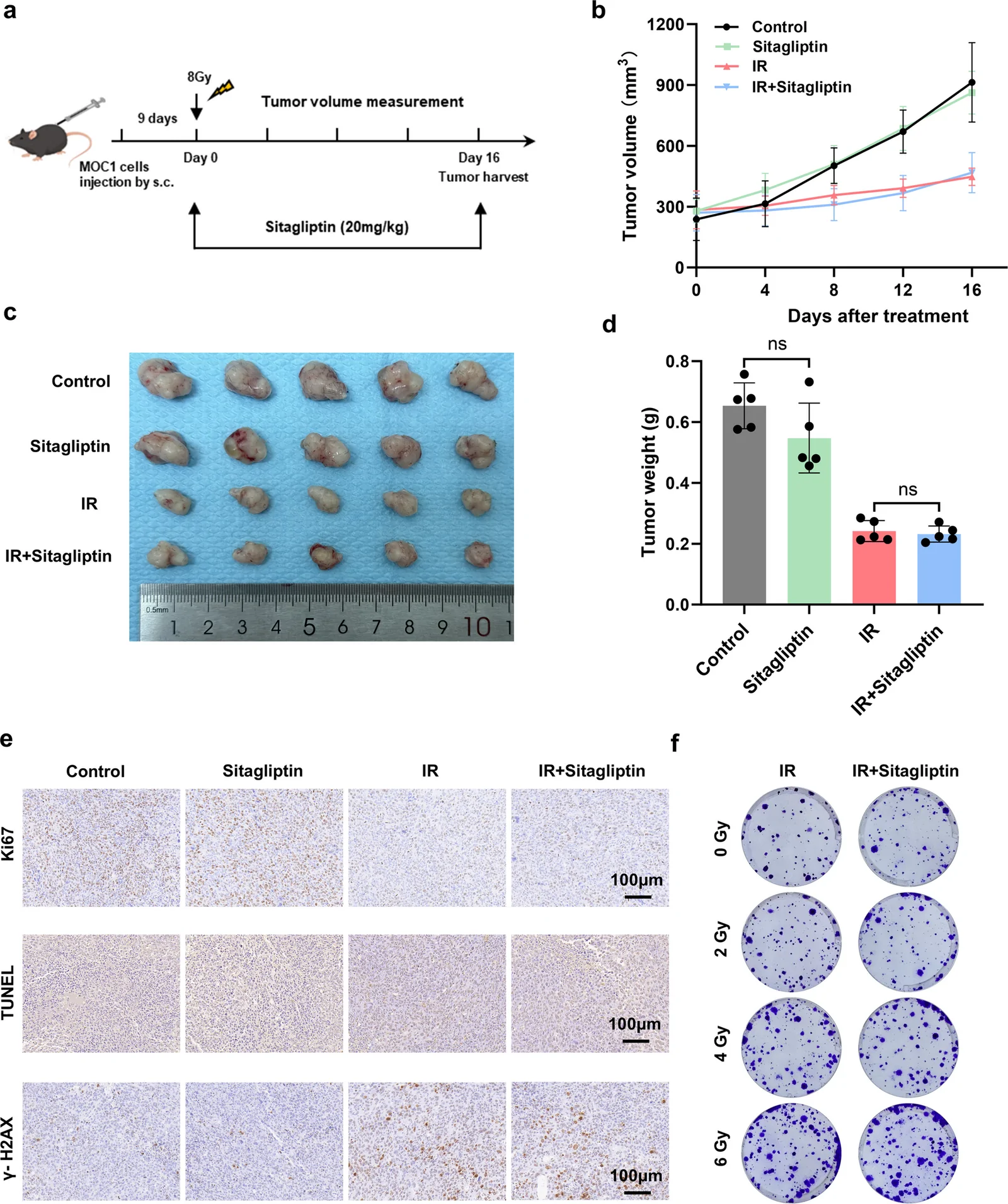
Sitagliptin does not compromise radiotherapy-induced tumor suppression. **a** Experimental design for the MOC1 tumor model. **b** Tumor growth curves (*n* = 5). **c**, **d** Macroscopic images and quantitative analysis of the tumor weight of the resected tumors (*n* = 5). **e** Ki67, TUNEL and γ-H2AX staining of tumor tissues. **f** Representative images of clonogenic survival assays in MOC1 cells irradiated at 0, 2, 4, or 6 Gy with or without sitagliptin treatment. Data are presented as mean ± SD. ns, not significant

To determine whether sitagliptin affects the antitumor efficacy of radiotherapy, a syngeneic subcutaneous tumor model was established using MOC1 cells. Tumor-bearing mice received localized irradiation (8 Gy) with or without sitagliptin treatment (Fig. 7a). Sitagliptin alone did not significantly affect tumor growth, whereas irradiation markedly suppressed tumor progression. Notably, tumor growth in the IR + Sitagliptin group was comparable to that in the IR group throughout the observation period (Fig. 7b). At day 16 post-treatment, tumors were collected for further analysis (Fig. 7c), and tumor weight was reduced to a similar extent in the IR and IR + Sitagliptin groups, with no statistically significant difference between the two groups (Fig. 7d). Consistent with these findings, Ki67, TUNEL, and γ-H2AX staining showed that irradiation reduced tumor-cell proliferation and increased apoptosis and DNA damage, whereas comparable staining patterns were observed between the IR and IR + Sitagliptin groups (Fig. 7e). To further assess tumor-cell radiosensitivity, clonogenic survival assays were performed in MOC1 cells following radiation doses of 0, 2, 4, and 6 Gy. Irradiation caused a dose-dependent reduction in clonogenic survival, while sitagliptin did not significantly alter the survival curves (Fig. 7f and Fig. S8). Together, these findings support the radioprotective potential of sitagliptin for oral mucosal injury without measurably compromising radiotherapy-induced tumor suppression.

**Fig. 8:**
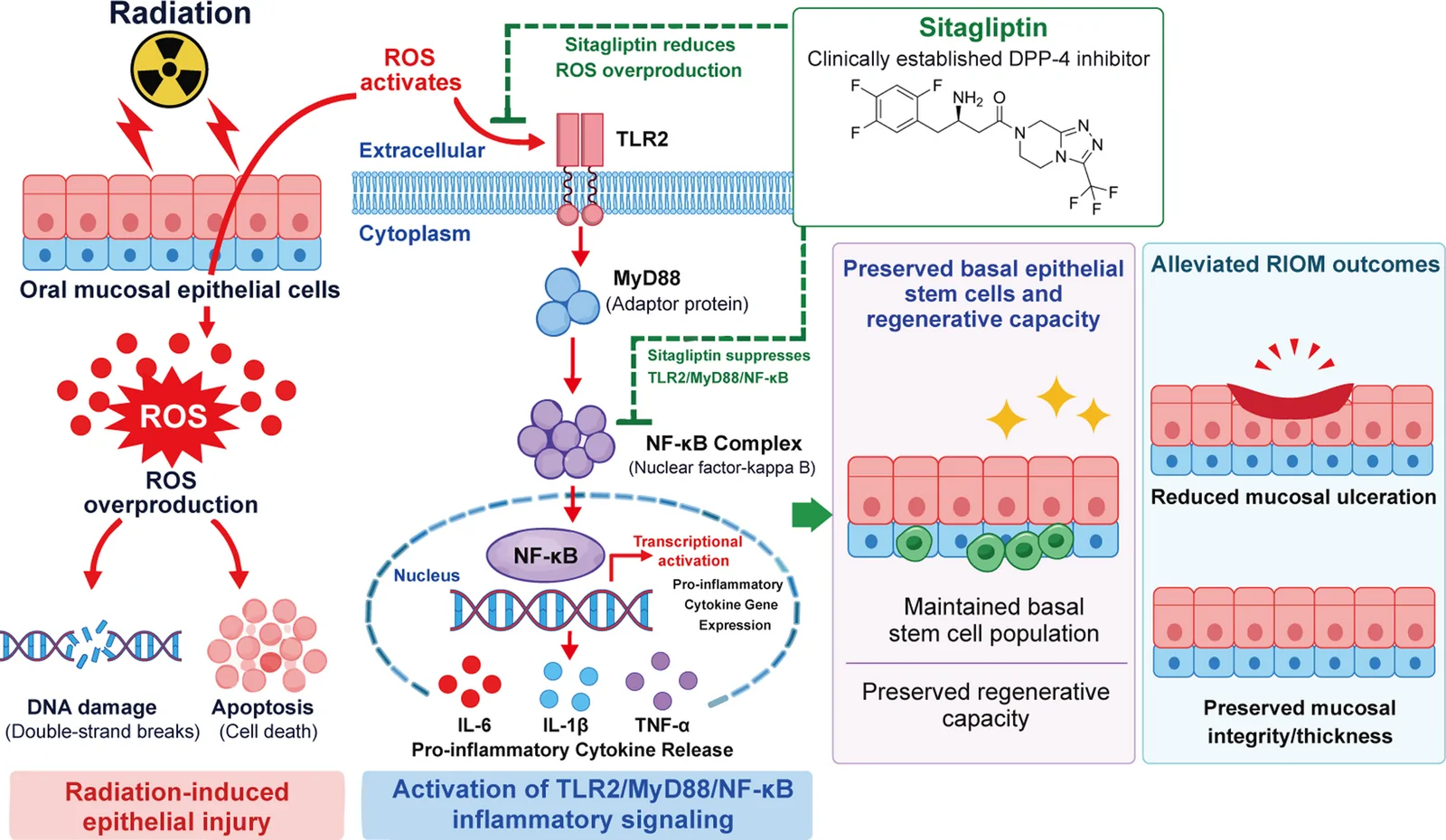
Schematic illustration of the mechanism by which sitagliptin alleviates radiation-induced oral mucositis. Radiation induces excessive ROS production, resulting in DNA damage and apoptosis and promoting activation of TLR2/MyD88/NF-κB inflammatory signaling and pro-inflammatory cytokine production. Sitagliptin, a clinically established DPP-4 inhibitor, attenuates oxidative injury and suppresses TLR2/MyD88/NF-κB signaling, thereby reducing epithelial damage and inflammatory responses. These effects contribute to preservation of basal epithelial stem cells and regenerative capacity, ultimately reducing mucosal ulceration and maintaining mucosal integrity. Red arrows indicate activation or induction, whereas green dashed lines indicate inhibitory effects of sitagliptin

A schematic summary integrating the major findings of this study is presented in Fig. 8, illustrating the proposed protective effects of sitagliptin against radiation-induced oral mucositis through attenuation of oxidative stress, apoptosis, and inflammatory responses via inhibition of the TLR2/MyD88/NF-κB signaling pathway.

## Discussion and conclusion

RIOM is a common complication of radiotherapy for head and neck malignancies and can substantially impair patients’ quality of life [25, 26]. Effective preventive and therapeutic interventions remain limited, and RIOM often necessitates treatment interruption or dose reduction, thereby compromising local tumor control [27]. The present study demonstrates that sitagliptin, an established DPP-4 inhibitor, markedly alleviates RIOM in murine models without compromising the antitumor efficacy of radiotherapy.

According to the widely accepted five-stage model of RIOM pathogenesis, radiation-induced injury evolves through a sequential cascade from initial cellular damage to downstream signal amplification. Within this framework, impaired epithelial regeneration is closely linked to depletion of basal epithelial stem cells, which are essential for continuous mucosal renewal. Radiation exposure damages these progenitor cells, disrupting epithelial turnover and ultimately leading to ulcer formation [7, 28, 29]. Our findings indicate that sitagliptin effectively counteracts this depletion, preserving the population of basal stem cells and maintaining their proliferative capacity. By safeguarding this critical regenerative compartment, sitagliptin helps sustain mucosal integrity, thereby reducing ulcer formation and limiting associated weight loss.

In the initiation stage, a rapid burst of ROS production represents a major early event in radiation-induced tissue injury. Ionizing radiation not only induces direct DNA strand breaks but also generates large quantities of ROS through water radiolysis within tissues. These reactive species damage cellular membranes via lipid peroxidation and attack nuclear DNA, resulting in DSB formation and the activation of apoptotic pathways in basal epithelial cells [30, 31]. In this study, sitagliptin treatment significantly restored redox balance in irradiated mucosa. By enhancing endogenous antioxidant defenses and reducing excessive ROS accumulation, sitagliptin attenuated radiation-induced oxidative stress. This antioxidant activity contributes to the preservation of genomic integrity, decreases DSB formation, and prevents excessive epithelial cell apoptosis.

As the pathological process progresses into the signal amplification and ulceration phases, local tissue injury provokes a robust inflammatory response. Transcriptomic analyses revealed marked alterations in TLR and NF-κB signaling following irradiation and sitagliptin treatment, implicating the TLR2/MyD88/NF-κB pathway in radiation-induced mucosal inflammation. TLR2, a key component of innate immune sensing, can be activated by elevated ROS levels and damage-associated molecular patterns (DAMPs) released from injured tissues [32]. Upon activation, TLR2 recruits the adaptor protein MyD88, which in turn activates NF-κB and drives the expression of inflammatory cytokines, including TNF-α, IL-1β, and IL-6. These cytokines not only exacerbate tissue injury directly but also establish a positive feedback loop that amplifies inflammatory signaling, thereby contributing to the progression of severe mucosal damage [33–36]. Previous studies have demonstrated that sitagliptin exerts anti-inflammatory effects across multiple disease contexts, including attenuation of diabetic nephropathy and cardiovascular injury through suppression of NF-κB signaling, as well as protection against acute tissue damage via inhibition of TLR-mediated pathways [37–39]. Consistent with these reports, our experimental results indicate that sitagliptin significantly downregulates key components of the inflammatory signaling cascade in irradiated tongue tissues and reduces inflammatory-cell infiltration within the injured mucosa. Importantly, in irradiated oral keratinocytes, pharmacological activation of TLR2 signaling with Pam3CSK4 reactivated downstream MyD88/NF-κB signaling and attenuated the protective and anti-inflammatory effects of sitagliptin. Together, these findings provide mechanistic evidence for the involvement of TLR2/MyD88/NF-κB signaling in the protective effects of sitagliptin and are consistent with a model in which suppression of this pathway contributes to the alleviation of radiation-induced oral mucosal injury.

An effective radioprotective agent must selectively shield normal tissues without diminishing tumor radiosensitivity [40, 41]. In agreement with this requirement, our results indicate that sitagliptin did not adversely affect radiotherapy-mediated tumor control in the MOC1 tumor-bearing mouse model. Furthermore, as sitagliptin is widely used as an antihyperglycemic agent for type 2 diabetes [42, 43], its application in non-diabetic populations raises concerns regarding potential hypoglycemia. However, the glucose-lowering effect of sitagliptin is glucose-dependent [44, 45], and our data showed that its administration did not cause abnormal changes in fasting blood glucose levels in healthy mice. In addition, comprehensive biochemical and histopathological evaluations revealed no detectable systemic toxicity. The combination of favorable safety, lack of an adverse effect on radiotherapy-mediated tumor control, and efficacy in mitigating mucosal injury provides strong support for the translational potential of sitagliptin in this context.

From a translational perspective, the 20 mg/kg dose used in the present study is also supported by interspecies dose scaling. The recommended clinical dose of sitagliptin is 100 mg once daily. Using conventional body-surface-area-based interspecies scaling, with Km values of 37 for humans and 3 for mice [46], a dose of 100 mg/day in a 60-kg adult corresponds to a mouse-equivalent dose of approximately 20.6 mg/kg, which closely matches the dose used in our study. This dose correspondence provides additional support for the clinical relevance of the selected dosing regimen.

This study has several limitations. First, the in vivo experiments were conducted exclusively in male mice; whether sex influences radiation responses or the protective efficacy of sitagliptin remains unclear. Second, although the 20 mg/kg dose was selected according to body-surface-area-based interspecies dose conversion, dose–response relationships were not systematically evaluated, and the optimal dosing regimen remains to be established. Third, although the single-dose 20 Gy model provides a robust and reproducible platform for investigating acute RIOM [31, 36, 47], it does not reproduce the conventional fractionation schedules used in clinical head and neck radiotherapy. We also evaluated the efficacy of sitagliptin under a fractionated irradiation regimen of 8 Gy × 3. In the single-dose irradiation model, sitagliptin was administered prophylactically before irradiation, whereas in the fractionated irradiation model, treatment was initiated post-irradiation. Sitagliptin was effective under both prophylactic and post-irradiation treatment schedules, indicating that its protective effect is not restricted to pre-irradiation administration and supporting its broader potential clinical applicability. Nevertheless, this experimental fractionation schedule remains distinct from conventional clinical radiotherapy. In addition, although pharmacological pathway reactivation provides mechanistic evidence supporting the involvement of TLR2/MyD88/NF-κB signaling in the protective effects of sitagliptin, the possibility that other signaling pathways also contribute to these effects cannot be excluded. Finally, the present study primarily focused on the acute phase of RIOM, and long-term mucosal healing and late radiation effects were not systematically assessed. Future studies incorporating both sexes, broader sitagliptin dose ranges, more clinically representative fractionation schedules, and extended follow-up will be important to further establish the therapeutic window, durability, and translational potential of sitagliptin.

Overall, the present findings support sitagliptin as a potential therapeutic candidate for RIOM. By attenuating oxidative stress and apoptosis and suppressing excessive inflammatory responses associated with TLR2/MyD88/NF-κB signaling, sitagliptin preserves the regenerative capacity of basal epithelial stem cells while not adversely affecting the antitumor efficacy of radiotherapy. Together with its established clinical use and favorable safety profile, these findings provide a preclinical basis for further investigation of sitagliptin as a potential intervention for radiation-induced oral mucosal injury.

## Materials and methods

### RIOM model establishment and sitagliptin treatment

Male C57BL/6 mice (7 weeks old) were obtained from Hubei Biont Biological Technology Co., Ltd. and housed under specific pathogen-free conditions. Before experimental intervention, mice were assigned to three experimental groups according to a computer-generated random-number sequence: non-irradiated controls (Control), irradiation plus vehicle (IR + Vehicle), and irradiation plus sitagliptin (IR + Sitagliptin). Following anesthesia with sodium pentobarbital, the bodies of the mice were shielded with lead blocks, with only the head and neck region exposed to irradiation. Ionizing radiation was delivered using an RS2000 Biological X-ray Irradiator (Rad Source Technologies, USA) operated at 160 kVp and 25 mA, with a dose rate of 1.9 Gy/min. During irradiation, the dose rate within the irradiation field was monitored using a 0.6 cm^3^ Farmer ionization chamber connected to a PTW UNIDOS dosimeter and remained constant throughout the procedure. Mice received either a single irradiation dose of 20 Gy or fractionated irradiation of 8 Gy once daily for three consecutive days (8 Gy × 3; total dose, 24 Gy). The day of single-dose irradiation or the first fraction of fractionated irradiation was designated as day 0. Control animals underwent identical handling without irradiation. For the single-dose irradiation experiment, sitagliptin (20 mg/kg) or saline was administered once daily by oral gavage beginning 5 days before irradiation and continuing through day 7. For the fractionated irradiation experiment, sitagliptin (20 mg/kg) or saline was first administered by oral gavage after completion of the third and final irradiation fraction and then administered once daily through day 7. The general condition and body weight of each mouse were monitored daily. On day 8, the mice were euthanized, and tissues were harvested for further analysis. All randomized mice and collected samples were included in the final analyses, and no exclusions were made.

### Macroscopic and histopathological analysis

Mouse tongues were collected after euthanasia, briefly rinsed in PBS, and immersed in 1% toluidine blue solution for 2 min. After washing with acetic acid, the tongues were photographed for macroscopic evaluation of ulcerative lesions. Whole-tongue images were analyzed using ImageJ software. The total tongue surface was manually delineated, and toluidine blue-positive areas were segmented using a fixed color threshold that was applied uniformly to all images within the same experiment. The percentage of mucosal damage was calculated as follows: toluidine blue-positive area/total tongue surface area × 100%.

For histological analysis, tongue specimens were subsequently fixed in 4% paraformaldehyde and processed for paraffin embedding. Sections (4 μm) were then prepared and stained with hematoxylin and eosin (H&E) to assess mucosal thickness and histopathological changes. Mucosal thickness was measured using ImageJ software as the perpendicular distance from the basement membrane to the epithelial surface in five randomly selected, non-overlapping fields per animal. Measurements from the five fields were averaged to obtain one value for each animal. Macroscopic and histological assessments were independently performed by two investigators blinded to group allocation.

### Immunohistochemistry and immunofluorescence staining

Paraffin sections of tongue tissue were deparaffinized and rehydrated, followed by antigen retrieval. After blocking, the sections were incubated overnight at 4 ℃ with primary antibodies against p63 (HUABIO, #ET1610-44), CK14 (Proteintech, #60320–1-Ig), Ki67 (Abcam, #ab15580), and γ-H2AX (CST, #9718). After washing, the sections were incubated with HRP-conjugated secondary antibodies. The immunoreactive signal was then visualized using 3,3’-diaminobenzidine (DAB), followed by hematoxylin counterstaining. For immunofluorescence staining, tongue sections were incubated with primary antibodies against F4/80 (Proteintech, #28463–1-AP), MPO (Proteintech, #22225–1-AP) and CD3 (Proteintech, #17617–1-AP), followed by appropriate fluorescent secondary antibodies. The images were analyzed using ImageJ software. The positive signal was identified using a fixed intensity threshold established separately for each marker and applied uniformly to all images within the same staining batch. Positive cells were quantified in five randomly selected, non-overlapping microscopic fields per animal, and the field-level measurements were averaged to obtain one value for each animal. The quantification was independently performed by two investigators blinded to group allocation.

### TUNEL assay

To evaluate the effects of irradiation and sitagliptin on mucosal cell apoptosis, tongue tissue sections were subjected to TUNEL staining using a TUNEL assay kit (Abbkine, China), according to the manufacturer’s instructions. Fluorescence or IHC images were acquired using identical exposure settings and analyzed using ImageJ software. A fixed fluorescence threshold was applied uniformly to all images within the same experiment, and TUNEL-positive cells were counted in five randomly selected, non-overlapping fields per animal. Measurements from the five fields were averaged to obtain one value for each animal. The quantification was independently performed by two investigators blinded to group allocation.

### DHE staining

ROS levels were measured in frozen tissue sections using dihydroethidium (DHE) staining (Solarbio, China), with nuclei counterstained using DAPI (Life-iLab, China). Fluorescence images were acquired using identical exposure and acquisition settings for all groups. Images were analyzed using ImageJ software, and background-subtracted mean DHE fluorescence intensity was measured in five randomly selected, non-overlapping fields per animal. A fixed fluorescence threshold was used to exclude background signals and was applied uniformly to all images within the same experiment. Measurements from the five fields were averaged to obtain one value for each animal. The quantification was independently performed by two investigators blinded to group allocation.

### MDA, GSH and SOD detection

MDA and GSH contents and SOD activity were quantified with commercially available kits (Servicebio, China) following the supplied protocols. Total protein was measured by the BCA method and used for normalization. The normalized values for MDA, GSH, and SOD were reported as nmol/mg protein, nmol/mg protein, and U/mg protein, respectively.

### Cell culture

MOC1 cells were cultured in DMEM/F12 medium containing 10% fetal bovine serum and 1% penicillin/streptomycin. Human oral keratinocytes (HOK) were maintained in a specialized keratinocyte medium. Both cell types were cultured at 37 °C in a humidified incubator with 5% CO_2_.

### Western blotting

Tongue tissues and cultured cells were lysed in RIPA buffer (Life-iLab, China), and the protein-containing supernatants were obtained after centrifugation. Equal amounts of protein were resolved by SDS-PAGE and transferred onto PVDF membranes. Following blocking in 5% non-fat milk, the membranes were incubated with the indicated primary antibodies, including β-actin (Proteintech, #66009–1-Ig), Bcl-2 (ABclonal, #A19693), Bax (ABclonal, #A19684), TLR2 (Proteintech, #66645–1-Ig), p65 (CST, #8242), MyD88 (Proteintech, #67969–1-Ig) and p-p65 (CST, #3033), which were diluted in primary antibody diluent (NCM Biotech, China) according to the manufacturers’ recommendations. Appropriate secondary antibodies were then applied, and protein bands were detected using a G:BOX Chemi X imaging system (Syngene, UK) with enhanced chemiluminescence (ECL) reagents.

### RNA sequencing

Tongue tissues from three mice in each group were processed for total RNA extraction, followed by library preparation. Sequencing was performed on an Illumina HiSeq platform by Bioyi Biotechnology Co., Ltd. (Wuhan, China), with a sequencing depth of approximately 6 Gb per sample. Raw reads were filtered using fastp by removing reads containing adapters, reads with more than 10% ambiguous bases (N), and reads in which more than 50% of bases had quality scores below Q20. The resulting clean reads were further evaluated using FastQC. Approximately 40.52–53.08 million clean reads were retained per sample. The Q20 and Q30 rates ranged from 98.32% to 98.82% and from 94.72% to 96.39%, respectively, and the GC content ranged from 47.96% to 50.06%. Clean reads were aligned to the mouse reference genome using HISAT2. The total mapping rates ranged from 98.09% to 98.55%, with 86.40–90.50% of reads uniquely mapped. All nine samples underwent library preparation and sequencing within the same batch; therefore, no additional batch correction was required. Sample consistency was evaluated by Pearson correlation and principal component analysis (PCA). Differentially expressed genes (DEGs) were identified using DESeq2 R package [48], with thresholds of |log_2_(fold change)| ≥ 1 and *p*-value ≤ 0.05. Gene Ontology (GO) and Kyoto Encyclopedia of Genes and Genomes (KEGG) enrichment analyses, together with Gene Set Enrichment Analysis (GSEA), were conducted using the clusterProfiler package [49].

### Quantitative Real-Time PCR (qPCR)

Total RNA from mouse tongue tissues and cultured cells was extracted with TRIzol (Life-iLab, China), followed by reverse transcription into cDNA using the HiScript IV All-in-One Ultra RT SuperMix for qPCR (R433, Vazyme Biotech Co.,Ltd, China). qPCR was subsequently carried out using SupRealQ Ultra Hunter SYBR qPCR Master Mix(U +) (Q713, Vazyme Biotech Co.,Ltd, China) on a Real-Time PCR system. Primer sequences are provided in Table S1.

### CCK-8 assay

HOK cells were plated into 96-well plates at 5 × 10^3^ cells per well. After attachment, cells were treated with the indicated concentrations of sitagliptin and/or Pam3CSK4 for 24 h to determine their cytotoxicity and appropriate working concentrations. Following the indicated drug treatments and/or irradiation, CCK-8 reagent (Life-iLab, China) was added, and absorbance was read at 450 nm using a microplate reader.

### EdU assay

HOK cells were plated in 96-well plates at 5 × 10^3^ cells per well. After treatment with sitagliptin alone or together with Pam3CSK4, the cells were immediately exposed to 8 Gy irradiation and then maintained for 24 h. Cell proliferation was evaluated with an EdU Cell Proliferation Kit with Alexa Fluor 555 (MeilunBio, China), and fluorescence images were captured under a fluorescence microscope.

### Intracellular ROS assay

HOK cells were plated in 6-well plates and allowed to adhere. The cells were treated with sitagliptin alone or together with Pam3CSK4, and immediately subjected to 8 Gy irradiation. After 24 h, intracellular ROS was detected using the fluorescent probe DCFH-DA (Solarbio, China). ROS-associated fluorescence was observed by fluorescence microscopy and quantified by flow cytometry.

### Apoptosis assay

HOK cells were plated in 6-well plates and allowed to adhere. The cells were then treated with sitagliptin alone or together with Pam3CSK4, followed immediately by 8 Gy irradiation. After 24 h, apoptosis was detected using an Annexin V-FITC/PI kit (KeyGEN BioTECH, China) and quantified by flow cytometry.

### Blood biochemical analysis

Blood samples were obtained from mice via retro-orbital collection. After centrifugation, serum was separated and submitted for biochemical analysis, which was performed by Servicebio Technology Co., Ltd. (Wuhan, China).

### Mouse oral squamous cell carcinoma syngeneic model

To establish the tumor model, C57BL/6 mice received a subcutaneous injection of 1 × 10^6^ MOC1 cells in the right flank. Measurable tumors were observed in all mice by day 9 after inoculation. At that time, tumor-bearing mice were stratified by baseline tumor volume and assigned to four groups according to a computer-generated random sequence: Control, Sitagliptin, IR, and IR + Sitagliptin. Under sodium pentobarbital anesthesia, mice assigned to the irradiated groups underwent localized 8 Gy irradiation of the right-flank tumor, while the rest of the body was protected with lead shielding. The irradiation day was defined as day 0. Sitagliptin (20 mg/kg) was given by oral gavage once daily from 1 h before irradiation until day 15, whereas the corresponding vehicle groups received the same volume of saline. Tumor length and width were recorded every four days, and tumor volume was calculated as length × width^2^/2. On day 16, mice were euthanized, and tumors collected for photography and weight measurement. All randomized mice and collected samples were included in the final analyses, and no exclusions were made.

### Clonogenic survival assay

MOC1 cells were plated in 6-well plates at 500, 1000, 2000, or 4000 cells per well and treated with vehicle or sitagliptin before exposure to irradiation at 0, 2, 4, or 6 Gy. Following irradiation, cells were cultured for 10–14 days. Colonies were then fixed and stained with crystal violet, and colonies containing more than 50 cells were counted. Surviving fractions were normalized to the plating efficiency of the corresponding non-irradiated controls, and survival curves were fitted using the linear-quadratic model.

### Statistical analysis

Statistical analyses were conducted using GraphPad Prism 10.1. Data are presented as mean ± standard deviation (SD). Comparisons between two groups were performed using Student’s t-test, whereas differences among multiple groups were analyzed using one-way ANOVA followed by Tukey’s post-hoc test. *P* < 0.05 was considered significant.

## Supplementary Information

Supplementary Material 1.

## Data Availability

RNA-Seq data were deposited into the National Center for Biotechnology Information (NCBI) under the BioProject accession number PRJNA1525669. The data are available at https://www.ncbi.nlm.nih.gov/bioproject/PRJNA1525669.
