# Supplementary material for: Sitagliptin ameliorates radiation-induced oral mucositis by inhibiting oxidative damage and inflammatory response via TLR2/MyD88/NF-κB signaling pathway
Source: Mol Biomed. 2026 Nov 9;7:242. doi: 10.1186/s43556-026-00653-8 (PMC13748224; doi:10.1186/s43556-026-00653-8)
Supplement: Supplementary file 1 [file 43556_2026_653_MOESM1_ESM.pdf]

Supplementary materials

Table S1. Sequences of primers used for experiments in this study.

Gene	Forward primer (5′–3′)	Reverse primer (5′–3′)
Mouse TNF-α	GGTGCCTATGTCTCAGCCTCTT	GCCATAGAACTGATGAGAGGGAG
Mouse IL-6	TACCACTTCACAAGTCGGAGGC	CTGCAAGTGCATCATCGTTGTTC
Mouse IL-1β	TGGACCTTCCAGGATGAGGACA	GTTCATCTCGGAGCCTGTAGTG
Mouse β-actin	GGCTGTATTCCCCTCCATCG	CCAGTTGGTAACAATGCCATGT
Human TNF-α	CTCTTCTGCCTGCTGCACTTTG	ATGGGCTACAGGCTTGTCACTC
Human IL-6	AGACAGCCACTCACCTCTTCAG	TTCTGCCAGTGCCTCTTTGCTG
Human IL-1β	CCACAGACCTTCCAGGAGAATG	GTGCAGTTCAGTGATCGTACAGG
Human β-actin	CACCATTGGCAATGAGCGGTTC	AGGTCTTTGCGGATGTCCACGT

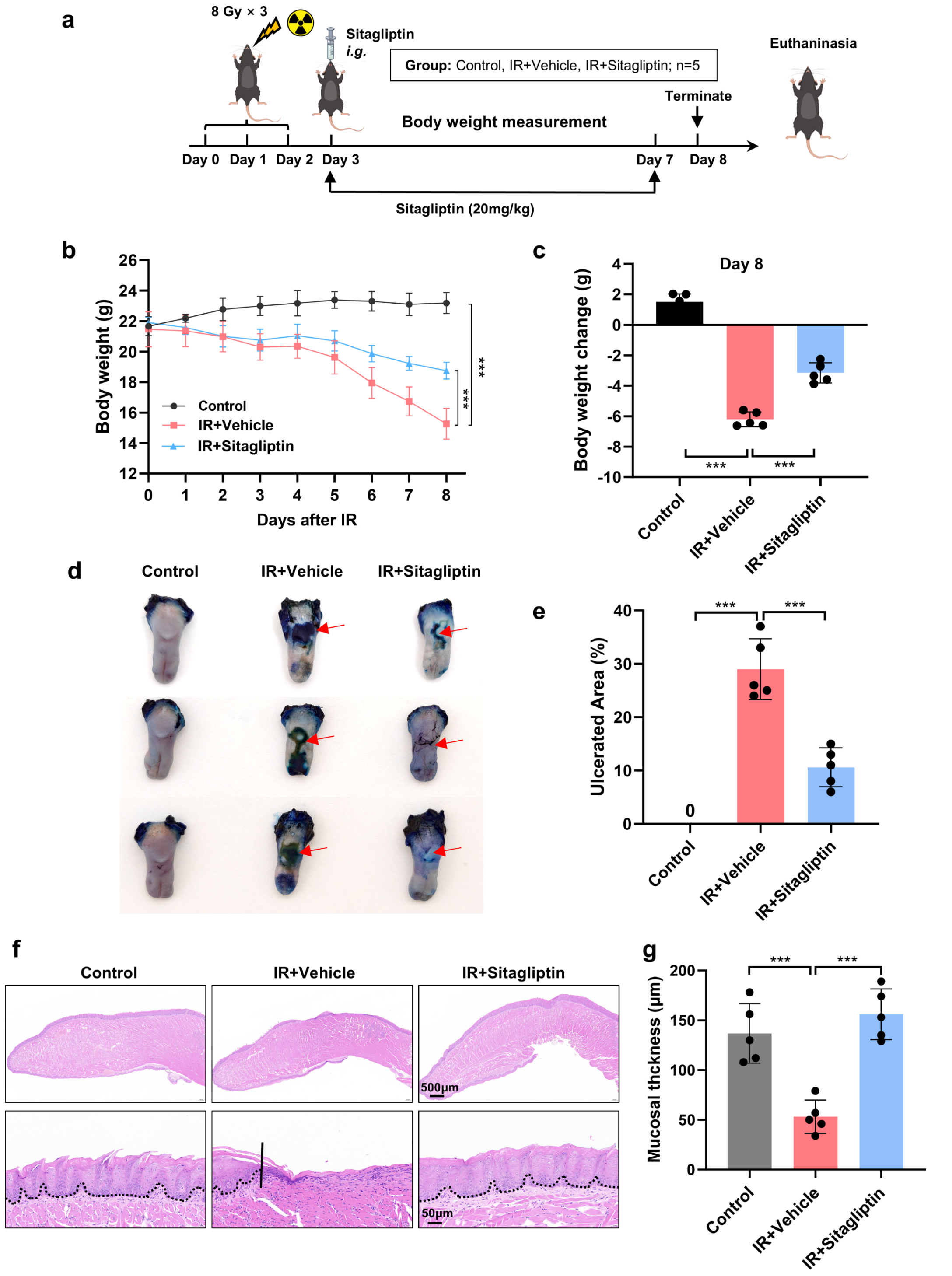

Figure S1. Sitagliptin alleviates radiation-induced oral mucositis under fractionated irradiation. (a) Experimental schematic of the fractionated irradiation regimen (8 Gy × 3) and post-irradiation sitagliptin treatment. (b) Body weight changes following irradiation. (c) Body weight change on day 8. (d) Representative images of toluidine blue-stained tongues; red arrows indicate ulcerative lesions. (e) Quantification of ulcerated surface area. (f) Representative H&E staining of mouse tongue mucosa. Vertical lines indicate ulcer boundaries, whereas dotted lines indicate the epithelial–stromal boundary. (g) Quantification of mucosal thickness. Data are presented as mean ± SD (n = 5 per group). ***$P < 0.001$.

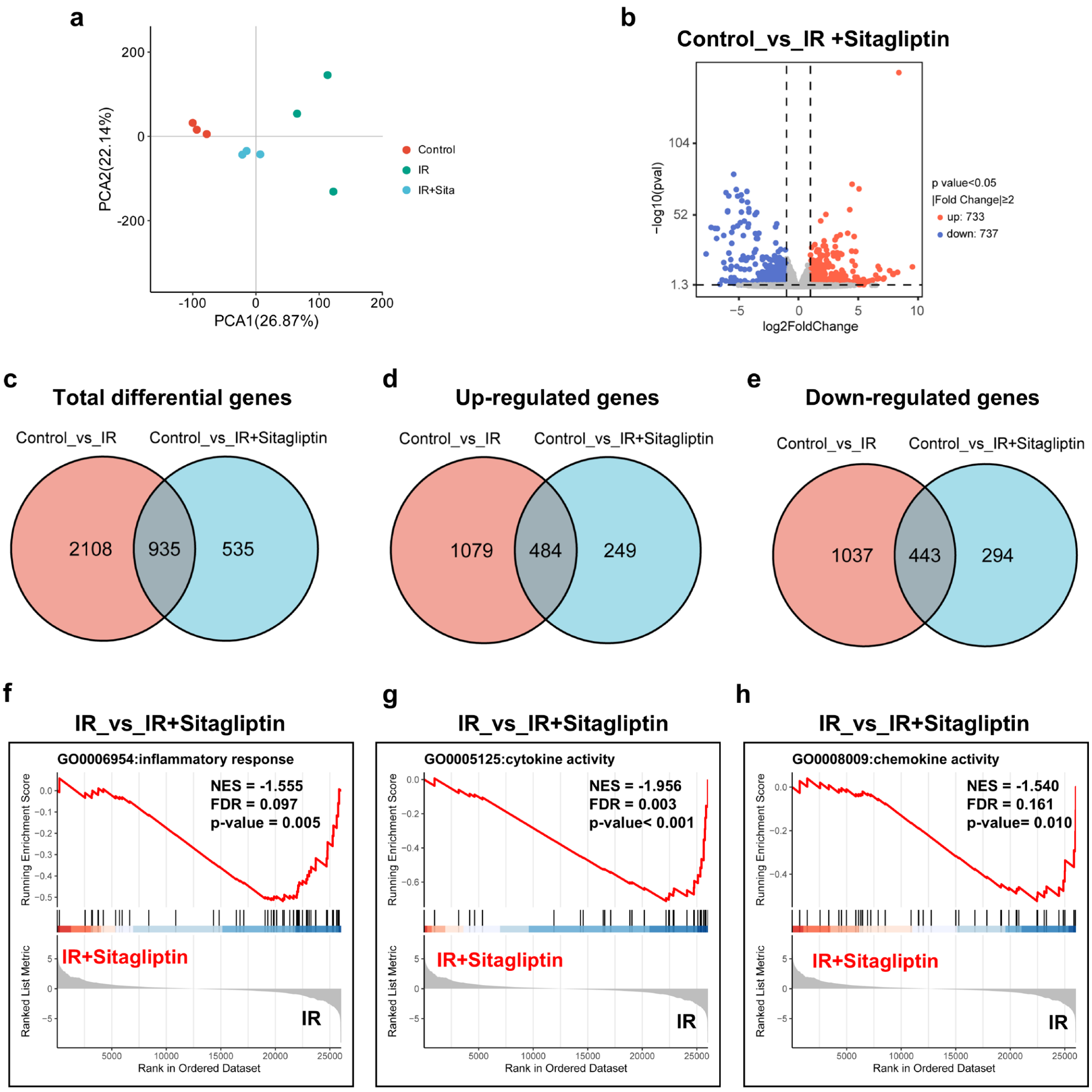

Figure S2. Reversal of radiation-induced transcriptomic alterations by sitagliptin and pathway enrichment analysis. (a) PCA analysis. (b) Volcano plot showing the DEGs between the Control and IR+Sitagliptin groups, including 733 upregulated and 737 downregulated genes. (c–e) Venn diagrams illustrating the overlap of total, up-regulated and down-regulated DEGs between the Control vs. IR and Control vs. IR+Sitagliptin comparisons. (f–h) GSEA showing significant suppression of the inflammatory response (NES = −1.555, FDR = 0.097, p-value = 0.005), cytokine activity (NES = −1.956, FDR = 0.003, p-value < 0.001), and chemokine activity (NES = −1.540, FDR = 0.161, p-value = 0.010) in the IR + Sitagliptin group compared with the IR group.

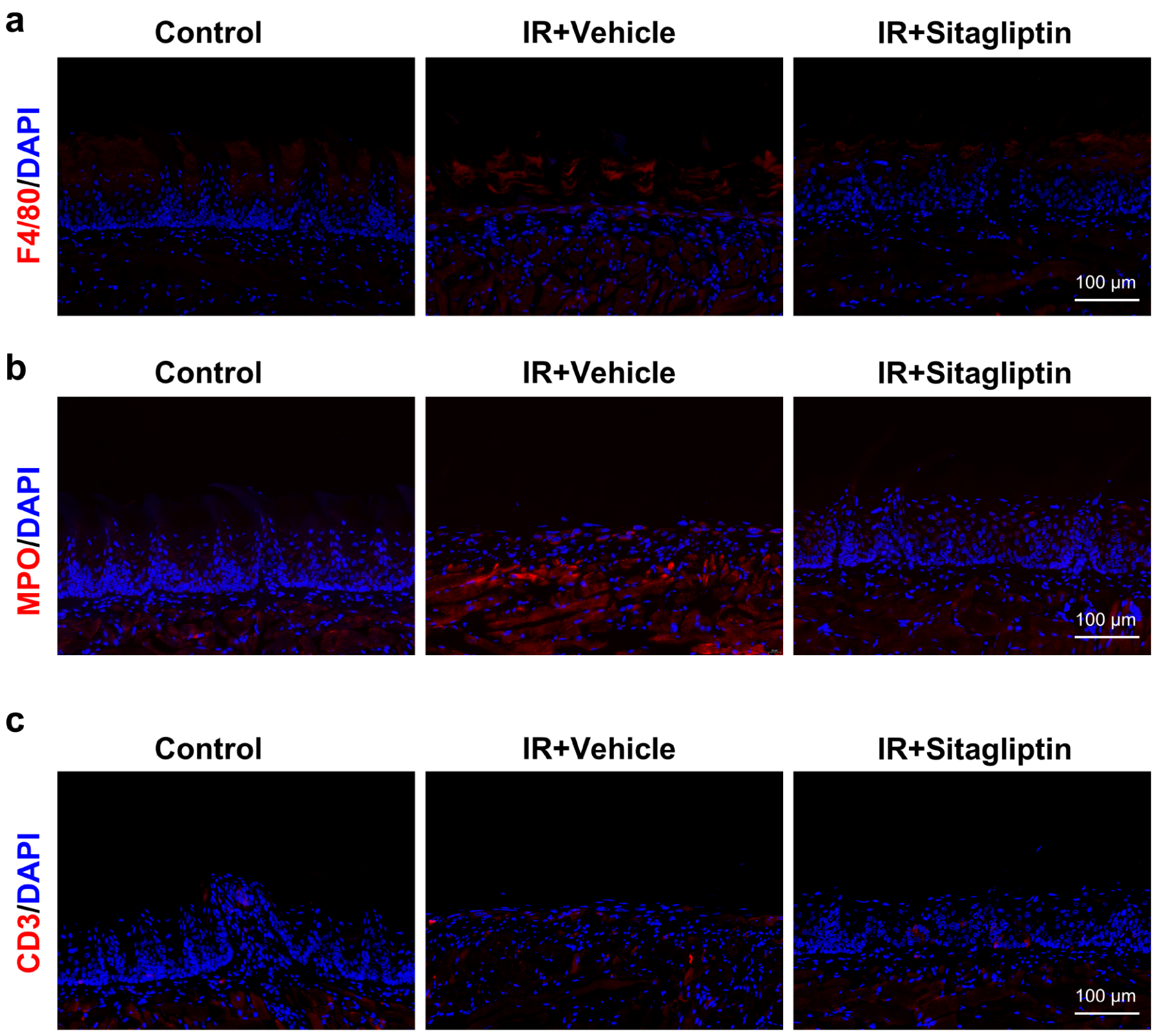

Figure S3. Immunofluorescence analysis of inflammatory-cell infiltration in mouse oral mucosa. Representative immunofluorescence staining of (a) F4/80-positive macrophages, (b) MPO-positive neutrophils, and (c) CD3-positive T cells in mouse tongue mucosal tissues.

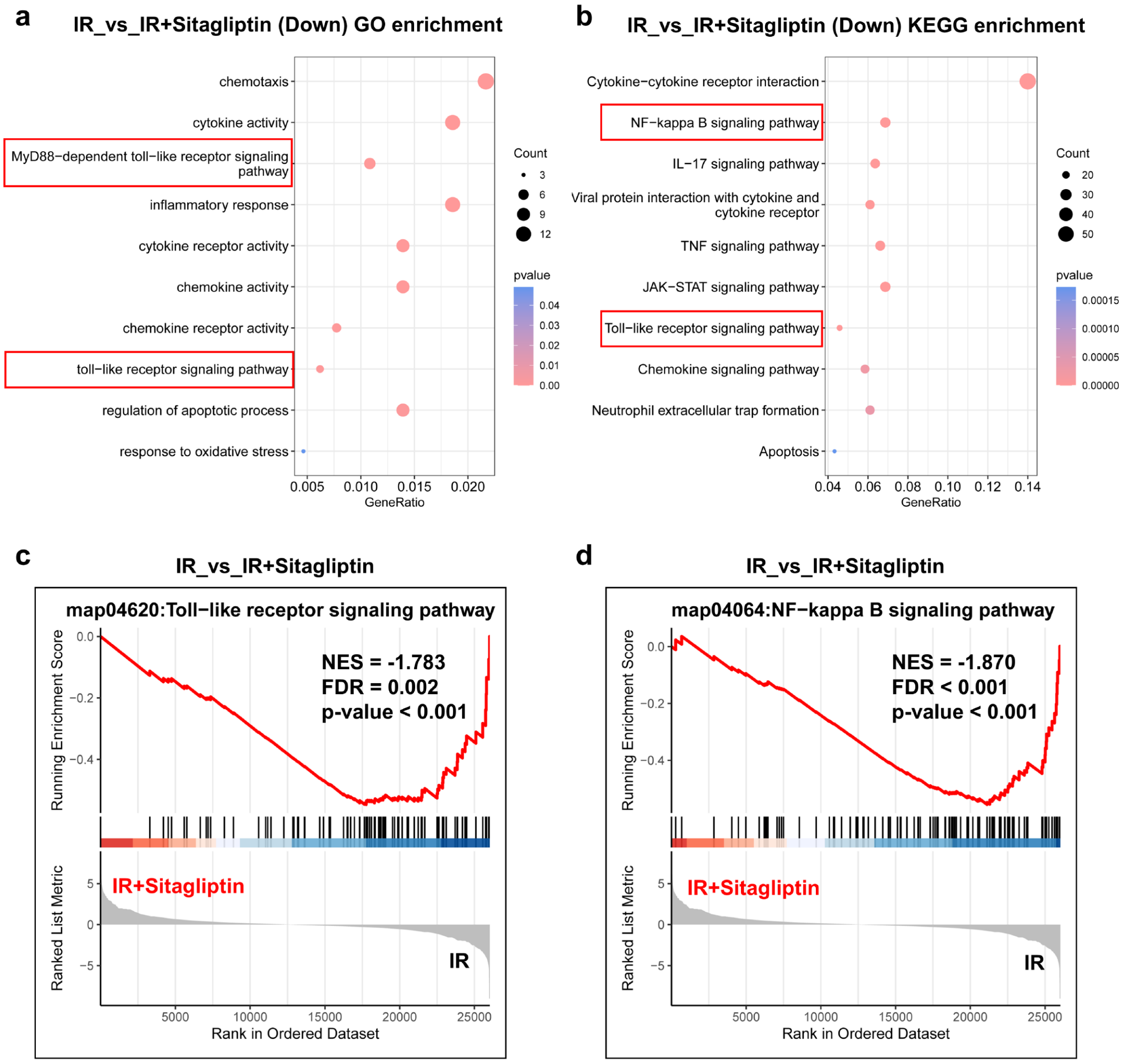

Figure S4. Sitagliptin significantly downregulates the radiation-activated Toll-like receptor and NF-κB signaling pathways. (a) GO enrichment analysis of downregulated DEGs in the IR + Sitagliptin group compared with the IR group. Red boxes highlight the MyD88-dependent Toll-like receptor signaling pathway (GeneRatio = 0.011, Count = 7, p-value = 3.19E-08) and Toll-like receptor signaling pathway (GeneRatio = 0.006, Count = 4, p-value = 0.0005). (b) KEGG enrichment analysis of downregulated DEGs in the IR + Sitagliptin group compared with the IR group. Red boxes highlight the NF-κB signaling pathway (GeneRatio = 0.069, Count = 27, p-value = 1.28E-13) and Toll-like receptor signaling pathway (GeneRatio = 0.046, Count = 18, p-value = 5.28E-07). (c, d) GSEA showing significant suppression of the Toll-like receptor signaling pathway (NES = −1.783, FDR = 0.002, p-value < 0.001) and NF-κB signaling pathway (NES = −1.870, FDR < 0.001, p-value < 0.001) in the IR + Sitagliptin group compared with the IR group.

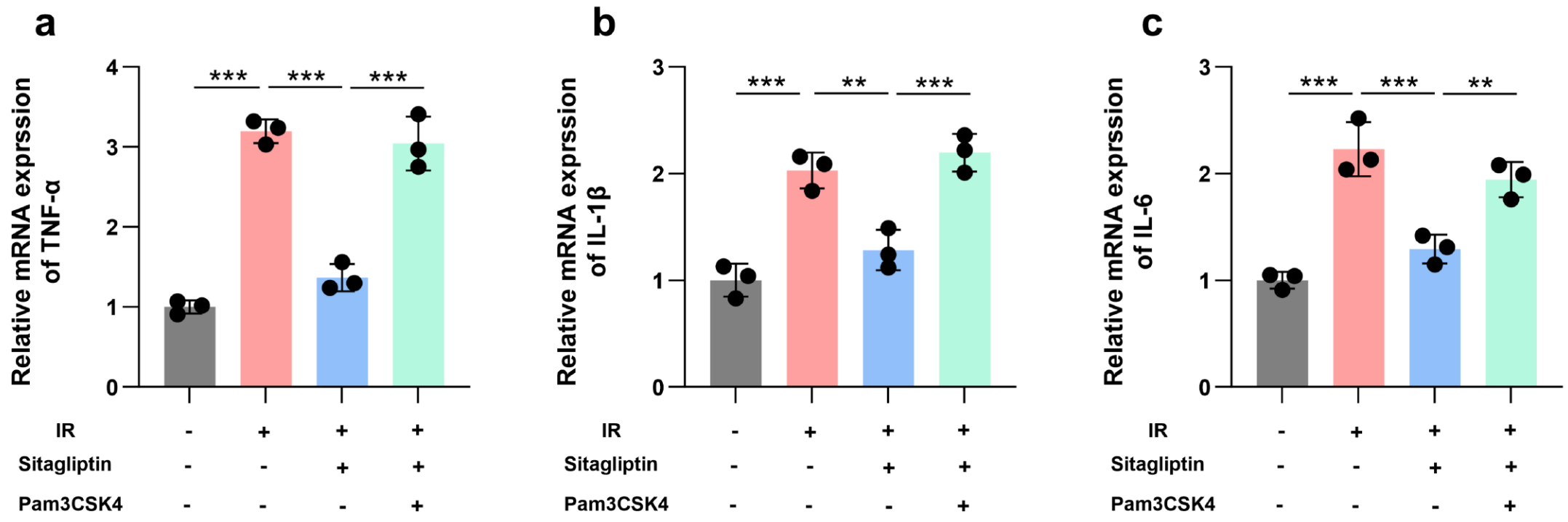

Figure S5. Effects of Pam3CSK4 on inflammatory cytokine expression in irradiated HOK cells. (a–c) RT-qPCR analysis of the relative mRNA expression levels of TNF-α (a), IL-1β (b), and IL-6 (c) in HOK cells under the indicated treatment conditions. Data are presented as mean ± SD from three independent experiments (n = 3). $^{**}P < 0.01$, $^{***}P < 0.001$.

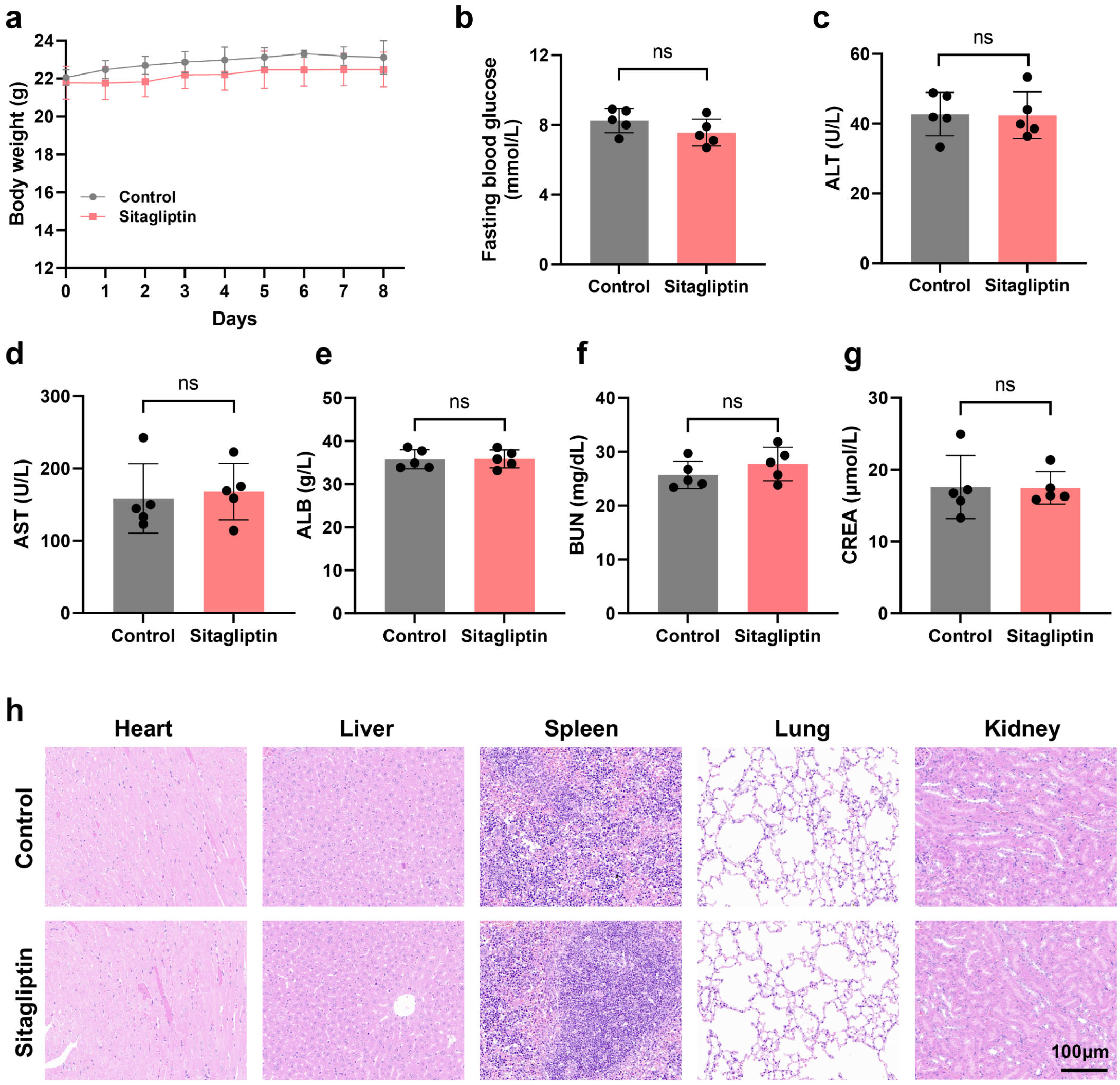

Figure S6. In vivo safety evaluation of sitagliptin. (a) Body weight changes during the 8-day observation period. (b) Fasting blood glucose levels. (c–g) Serum biochemical parameters (ALT, AST, ALB, BUN, and CREA). (h) Representative H&E staining of major organs. Data are presented as mean ± SD (n = 5 per group). ns, not significant.

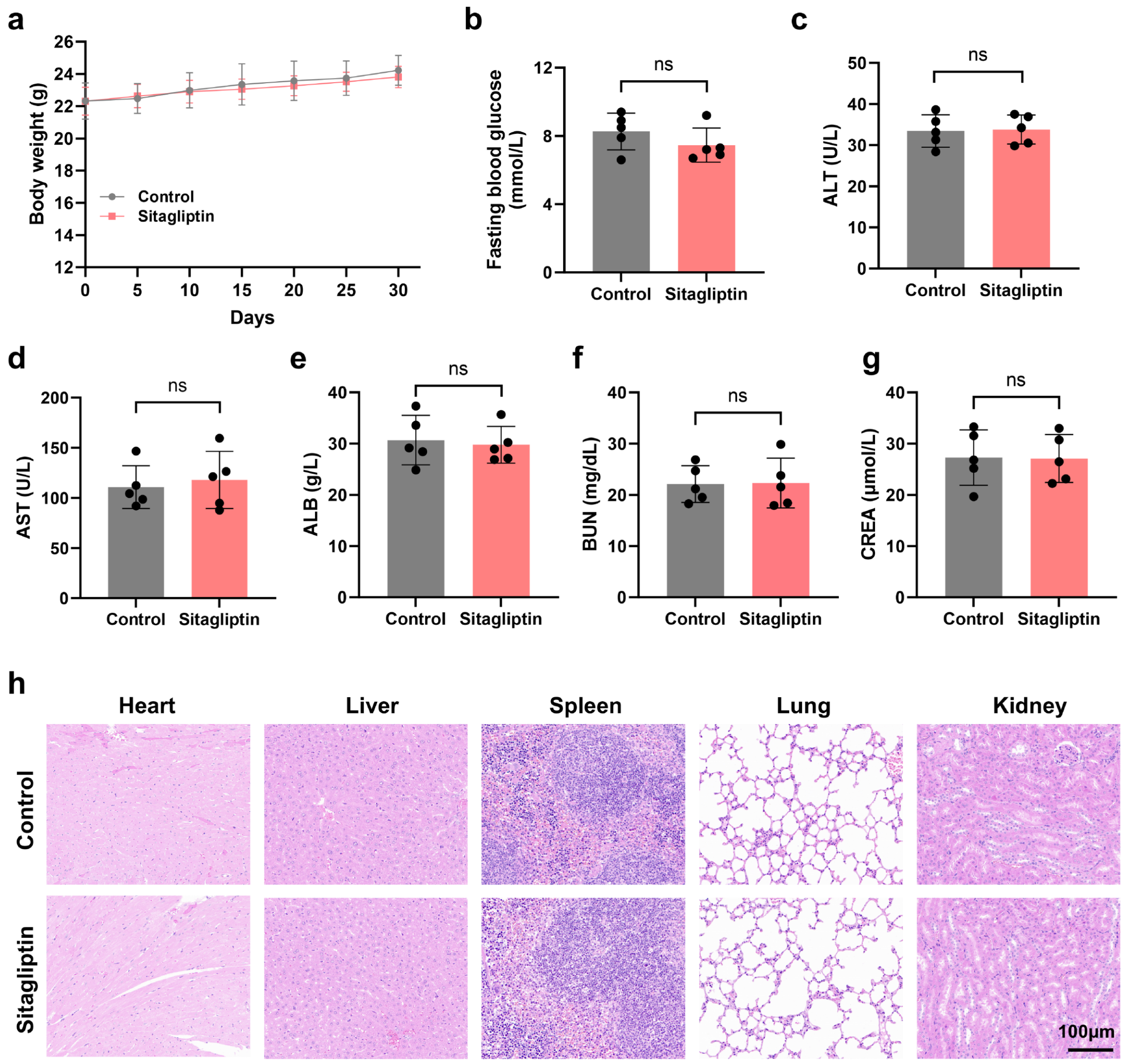

Figure S7. Long-term in vivo safety evaluation of sitagliptin. (a) Body weight changes during the 30-day observation period. (b) Fasting blood glucose levels. (c–g) Serum biochemical parameters (ALT, AST, ALB, BUN, and CREA). (h) Representative H&E staining of major organs. Data are presented as mean ± SD (n = 5 per group). ns, not significant.

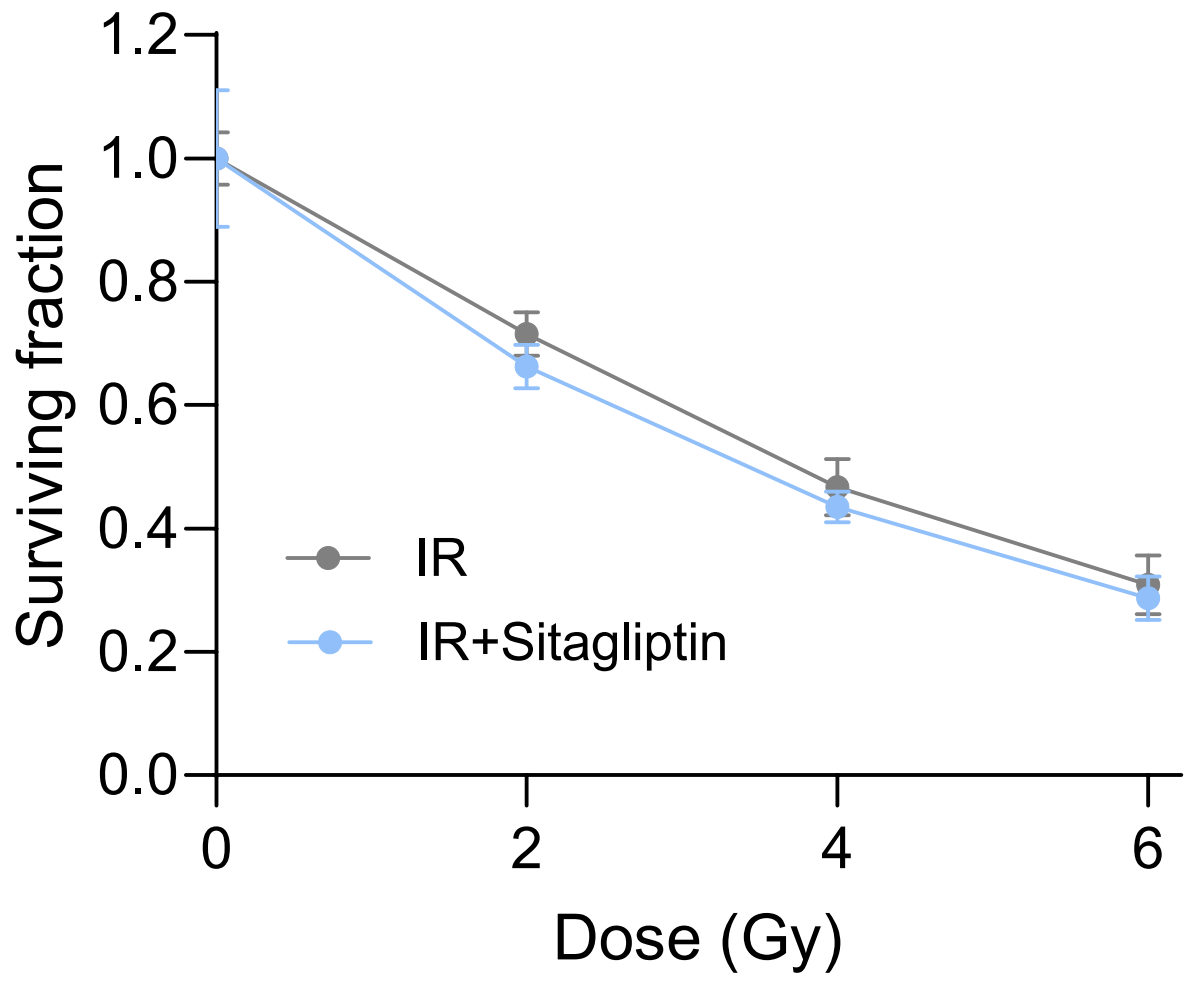

Figure S8. Clonogenic survival of MOC1 cells following irradiation. Clonogenic survival curves of MOC1 cells exposed to 0, 2, 4, and 6 Gy irradiation with or without sitagliptin treatment. Data are presented as mean ± SD (n = 3 independent experiments).
